# Supplementary material for: A Comparative Study of New Fluorescent Anthraquinone and Benzanthrone α-Aminophosphonates: Synthesis, Spectroscopy, Toxicology, X-ray Crystallography, and Microscopy of Opisthorchis felineus
Source: Molecules. 2024 Mar 4;29(5):1143. doi: 10.3390/molecules29051143 (PMC10934897; doi:10.3390/molecules29051143)
Supplement: Supplementary file 1 [file molecules-29-01143-s001.zip › Supplementary Material.pdf]

Supplementary Material

A comparative study of new fluorescent anthraquinone and benzanthrone α-aminophosphonates: synthesis, spectroscopy, X-ray crystallography and microscopy of *Opisthorchis felineus*

Armands Maļeckis [1,*], Marija Cvetinska [1], Muza Kirjušina [2], Ligita Mežaraupe [2], Sanita Kecko [2], Inese Gavarāne [2], Vladimir Kiyan [3], Lyudmila Lider [4], Veronika Pavlova [2], Marina Savicka [2], Sergey Belyakov [5] and Elena Kirilova [6]

[1] Institute of Chemistry and Chemical Technology, Faculty of Natural Sciences and Technology, Riga Technical University, P. Valdena Str. 3, LV-1048 Riga, Latvia
[2] Department of Ecology, Institute of Life Sciences and Technology, Daugavpils University, LV-5401 Daugavpils, Latvia
[3] Laboratory of Biodiversity and Genetic Resources, National Center for Biotechnology, 13/5 Kurgalzhynskoye road, 010000 Astana, Kazakhstan
[4] S. Seifullin Kazakh Agro Technical Research University, Faculty of Veterinary Medicine and Animal Husbandry Technology, 62 Zhenis Avenue, 010011 Astana, Kazakhstan
[5] Latvian Institute of Organic Synthesis, Aizkraukles Str. 21, LV-1006 Riga, Latvia; serg@osi.lv
[6] Department of Environment and Technologies, Faculty of Natural Sciences and Healthcare, Daugavpils University, LV-5401 Daugavpils, Latvia
* Correspondence: armands5maleckis@inbox.lv

Table S1. Crystal data and structure refinement for 2a.

Identification code	V2
Empirical formula	$C_{23}H_{19}BrNO_5P$
Formula weight	500.27
Temperature/K	150.0(3)
Crystal system	triclinic
Space group	$P\bar{1}$
a/Å	7.56264(18)
b/Å	7.78764(18)
c/Å	18.0152(5)
α/°	85.582(2)
β/°	78.626(2)
γ/°	85.6433(19)
Volume/Å^3	1035.07(5)
Z	2
ρ_{calc}g/cm^3	1.605
μ/mm^{-1}	3.759
F(000)	508.0
Crystal size/mm^3	0.22 × 0.13 × 0.06
Radiation	CuKα (λ = 1.54184 Å)
2Θ max. for data collection/°	160
Index ranges	$-9 \le h \le 8$, $-9 \le k \le 9$, $-23 \le l \le 22$
Reflections collected	17799
Independent reflections	4456 [R_{int} = 0.0343, R_{sigma} = 0.0246]
Data/restraints/parameters	4456/0/287
Goodness-of-fit on F^2	1.099
Final *R* indexes [$I > 2\sigma(I)$]	R_1 = 0.0770, wR_2 = 0.2013
Final *R* indexes [all data]	R_1 = 0.0781, wR_2 = 0.2016
Largest diff. peak/hole / e Å^{-3}	1.87/-0.57

Table S2. Fractional Atomic Coordinates (×10[4]) and Equivalent Isotropic Displacement Parameters (Å[2]×10[3]) for 2a. U_{eq} is defined as 1/3 of of the trace of the orthogonalised U_{IJ} tensor.

**Atom**	***x***	***y***	***z***	***U*(eq)**
C1	5307(8)	1417(8)	3416(3)	24.3(12)
C2	3712(8)	913(8)	3212(4)	26.2(12)
C3	2136(8)	793(8)	3741(4)	28.9(13)
C4	2070(8)	1192(8)	4482(4)	27.7(13)
C4A	3578(7)	1736(7)	4699(3)	23.4(11)
C5	4827(9)	3485(8)	6444(4)	31.6(14)
C6	6277(9)	4177(8)	6649(4)	30.5(13)
C7	7878(9)	4324(8)	6134(4)	30.0(13)
C8	8023(8)	3767(8)	5408(4)	28.0(12)
C8A	6577(8)	3101(7)	5186(3)	24.4(12)
C9	6772(8)	2556(8)	4398(3)	25.0(12)
C9A	5231(7)	1869(7)	4169(3)	23.0(11)
C10	3395(8)	2234(8)	5494(3)	26.9(12)
C10A	4948(8)	2956(8)	5718(3)	25.4(12)
N11	6917(7)	1440(7)	2895(3)	27.0(11)
C12	6987(8)	1412(8)	2088(3)	27.4(12)
P13	9322(2)	810(2)	1648.9(10)	30.9(4)
O14	10651(6)	1781(7)	1894(3)	39.6(12)
O15	9510(7)	-1205(7)	1790(3)	42.2(12)
C16	10495(10)	-2042(10)	2329(4)	40.7(16)
O17	9286(6)	957(7)	776(3)	38.0(11)
C18	10100(13)	2323(11)	272(5)	53(2)
C19	6322(8)	3106(8)	1727(3)	24.7(12)
C20	4817(9)	3202(9)	1384(4)	32.0(14)
C21	4204(10)	4751(9)	1066(4)	38.0(15)
C22	5100(9)	6210(9)	1095(4)	33.9(14)
C23	6584(9)	6169(9)	1433(4)	36.7(15)
C24	7179(9)	4614(9)	1757(4)	32.8(14)
Br25	4271.0(12)	8345.6(10)	655.3(5)	47.2(3)
O26	8238(5)	2743(6)	3964(2)	32.5(10)
O27	1971(6)	2077(7)	5947(3)	38.5(11)

Table S3. Anisotropic Displacement Parameters (Å^2×10^3) for 2a. The Anisotropic displacement factor exponent takes the form: $-2\pi^2[h^2a^{*2}U_{11}+2hka^*b^*U_{12}+\ldots]$.

**Atom**	U_{11}	U_{22}	U_{33}	U_{23}	U_{13}	U_{12}
C1	19(3)	26(3)	27(3)	0(2)	-4(2)	-4(2)
C2	20(3)	30(3)	28(3)	-4(2)	-5(2)	-1(2)
C3	21(3)	28(3)	39(3)	-1(3)	-8(2)	-3(2)
C4	18(3)	27(3)	36(3)	0(2)	-1(2)	-3(2)
C4A	18(3)	24(3)	27(3)	2(2)	-2(2)	2(2)
C5	31(3)	32(3)	31(3)	-1(3)	-4(3)	1(3)
C6	37(3)	29(3)	25(3)	-6(2)	-4(3)	5(3)
C7	29(3)	29(3)	33(3)	-1(3)	-10(3)	1(2)
C8	27(3)	29(3)	28(3)	-2(2)	-6(2)	-4(2)
C8A	21(3)	23(3)	28(3)	2(2)	-4(2)	2(2)
C9	20(3)	25(3)	29(3)	1(2)	-5(2)	2(2)
C9A	19(3)	21(3)	28(3)	1(2)	-5(2)	-1(2)
C10	23(3)	27(3)	28(3)	3(2)	-3(2)	5(2)
C10A	22(3)	24(3)	29(3)	1(2)	-4(2)	3(2)
N11	23(2)	34(3)	25(3)	-4(2)	-4(2)	-6(2)
C12	23(3)	35(3)	25(3)	-7(2)	-3(2)	-5(2)
P13	22.8(8)	38.3(9)	32.4(8)	-12.1(7)	-3.0(6)	-3.9(6)
O14	25(2)	49(3)	45(3)	-17(2)	-1(2)	-7(2)
O15	33(3)	43(3)	56(3)	-12(2)	-20(2)	3(2)
C16	36(4)	51(4)	34(4)	-3(3)	-5(3)	-3(3)
O17	31(2)	48(3)	34(3)	-15(2)	2.3(19)	-9(2)
C18	68(6)	51(5)	38(4)	-6(4)	2(4)	-16(4)
C19	24(3)	30(3)	19(3)	-8(2)	0(2)	-4(2)
C20	34(3)	30(3)	34(3)	-2(3)	-9(3)	-7(3)
C21	37(4)	39(4)	42(4)	-2(3)	-16(3)	-2(3)
C22	34(3)	32(3)	31(3)	7(3)	0(3)	-2(3)
C23	34(3)	31(3)	43(4)	-9(3)	3(3)	-9(3)
C24	27(3)	41(4)	32(3)	-10(3)	-5(3)	-6(3)
Br25	52.7(5)	34.1(4)	48.6(5)	7.2(3)	0.0(4)	2.1(3)
O26	17(2)	51(3)	29(2)	-6(2)	0.1(17)	-5.9(19)
O27	22(2)	62(3)	29(2)	-6(2)	3.4(18)	-5(2)

Table S4. Bond Lengths for 2a.

Atom	Atom	Length/Å	Atom	Atom	Length/Å
C1	C2	1.419(8)	C10	C10A	1.476(9)
C1	C9A	1.417(8)	C10	O27	1.223(7)
C1	N11	1.384(7)	N11	C12	1.446(8)
C2	C3	1.376(8)	C12	P13	1.828(6)
C3	C4	1.384(9)	C12	C19	1.520(9)
C4	C4A	1.381(8)	P13	O14	1.456(5)
C4A	C9A	1.421(8)	P13	O15	1.570(6)
C4A	C10	1.490(8)	P13	O17	1.573(5)
C5	C6	1.381(10)	O15	C16	1.427(9)
C5	C10A	1.386(9)	O17	C18	1.438(9)
C6	C7	1.379(9)	C19	C20	1.393(9)
C7	C8	1.392(9)	C19	C24	1.393(9)
C8	C8A	1.384(8)	C20	C21	1.383(10)
C8A	C9	1.491(8)	C21	C22	1.375(10)
C8A	C10A	1.409(8)	C22	C23	1.376(10)
C9	C9A	1.460(8)	C22	Br25	1.903(7)
C9	O26	1.235(7)	C23	C24	1.385(10)

Table S5. Bond Angles for V2.

Atom	Atom	Atom	Angle/˚	Atom	Atom	Atom	Angle/˚
C9A	C1	C2	118.8(5)	C5	C10A	C8A	119.6(6)
N11	C1	C2	120.8(5)	C5	C10A	C10	120.8(6)
N11	C1	C9A	120.4(5)	C8A	C10A	C10	119.6(6)
C3	C2	C1	120.8(6)	C1	N11	C12	122.3(5)
C2	C3	C4	120.5(6)	N11	C12	P13	107.3(4)
C4A	C4	C3	120.6(6)	N11	C12	C19	113.8(5)
C4	C4A	C9A	120.6(6)	C19	C12	P13	111.2(4)
C4	C4A	C10	118.0(5)	O14	P13	C12	113.7(3)
C9A	C4A	C10	121.4(5)	O14	P13	O15	116.5(3)
C6	C5	C10A	120.7(6)	O14	P13	O17	116.7(3)
C7	C6	C5	120.2(6)	O15	P13	C12	104.6(3)
C6	C7	C8	119.5(6)	O15	P13	O17	100.1(3)
C8A	C8	C7	121.2(6)	O17	P13	C12	103.4(3)
C8	C8A	C9	119.6(5)	C16	O15	P13	122.8(5)
C8	C8A	C10A	118.8(6)	C18	O17	P13	122.5(5)
C10A	C8A	C9	121.6(5)	C20	C19	C12	121.2(5)
C9A	C9	C8A	119.1(5)	C24	C19	C12	120.2(6)
O26	C9	C8A	117.6(5)	C24	C19	C20	118.6(6)
O26	C9	C9A	123.3(6)	C21	C20	C19	120.9(6)
C1	C9A	C4A	118.7(5)	C22	C21	C20	119.0(6)
C1	C9A	C9	121.6(5)	C21	C22	C23	121.8(6)
C4A	C9A	C9	119.6(5)	C21	C22	Br25	119.3(5)
C10A	C10	C4A	118.7(5)	C23	C22	Br25	118.9(5)
O27	C10	C4A	120.4(6)	C22	C23	C24	118.8(6)
O27	C10	C10A	120.9(6)	C23	C24	C19	120.9(6)

Table S6. Torsion Angles for 2a.

A	B	C	D	Angle/˚	A	B	C	D	Angle/˚
C1	C2	C3	C4	1.1(9)	C10A	C8A	C9	C9A	0.6(8)
C1	N11	C12	P13	-163.3(5)	C10A	C8A	C9	O26	179.2(6)
C1	N11	C12	C19	73.2(7)	N11	C1	C2	C3	176.2(6)
C2	C1	C9A	C4A	2.4(8)	N11	C1	C9A	C4A	-176.5(5)
C2	C1	C9A	C9	-174.0(5)	N11	C1	C9A	C9	7.1(9)
C2	C1	N11	C12	16.8(9)	N11	C12	P13	O14	-48.3(5)
C2	C3	C4	C4A	0.8(9)	N11	C12	P13	O15	79.9(5)
C3	C4	C4A	C9A	-1.1(9)	N11	C12	P13	O17	-175.8(4)
C3	C4	C4A	C10	176.9(6)	N11	C12	C19	C20	-117.7(6)
C4	C4A	C9A	C1	-0.6(8)	N11	C12	C19	C24	60.3(7)
C4	C4A	C9A	C9	176.0(5)	C12	P13	O15	C16	-106.7(5)
C4	C4A	C10	C10A	-175.0(5)	C12	P13	O17	C18	107.7(6)
C4	C4A	C10	O27	3.6(9)	C12	C19	C20	C21	179.2(6)
C4A	C10	C10A	C5	178.1(5)	C12	C19	C24	C23	-179.8(6)
C4A	C10	C10A	C8A	-2.2(8)	P13	C12	C19	C20	121.0(5)
C5	C6	C7	C8	-0.2(10)	P13	C12	C19	C24	-61.0(6)
C6	C5	C10A	C8A	1.0(9)	O14	P13	O15	C16	19.7(7)
C6	C5	C10A	C10	-179.3(6)	O14	P13	O17	C18	-17.9(7)
C6	C7	C8	C8A	1.4(9)	O15	P13	O17	C18	-144.5(6)
C7	C8	C8A	C9	178.6(6)	O17	P13	O15	C16	146.5(5)
C7	C8	C8A	C10A	-1.5(9)	C19	C12	P13	O14	76.7(5)
C8	C8A	C9	C9A	-179.5(5)	C19	C12	P13	O15	-155.1(4)
C8	C8A	C9	O26	-0.8(8)	C19	C12	P13	O17	-50.8(5)
C8	C8A	C10A	C5	0.3(9)	C19	C20	C21	C22	-0.2(11)
C8	C8A	C10A	C10	-179.5(5)	C20	C19	C24	C23	-1.8(9)
C8A	C9	C9A	C1	176.6(5)	C20	C21	C22	C23	-0.1(11)
C8A	C9	C9A	C4A	0.1(8)	C20	C21	C22	Br25	179.7(5)
C9	C8A	C10A	C5	-179.8(5)	C21	C22	C23	C24	-0.5(10)
C9	C8A	C10A	C10	0.5(8)	C22	C23	C24	C19	1.4(10)
C9A	C1	C2	C3	-2.7(9)	C24	C19	C20	C21	1.2(10)
C9A	C1	N11	C12	-164.4(6)	Br25	C22	C23	C24	179.7(5)
C9A	C4A	C10	C10A	2.9(8)	O26	C9	C9A	C1	-2.0(9)
C9A	C4A	C10	O27	-178.5(6)	O26	C9	C9A	C4A	-178.4(6)
C10	C4A	C9A	C1	-178.4(5)	O27	C10	C10A	C5	-0.5(9)
C10	C4A	C9A	C9	-1.9(8)	O27	C10	C10A	C8A	179.2(6)
C10A	C5	C6	C7	-1.0(10)					

Table S7. Hydrogen Atom Coordinates (Å×10^4) and Isotropic Displacement Parameters (Å^2×10^3) for 2a.

**Atom**	***x***	***y***	***z***	***U*(iso)**
H2	3731.89	655.22	2702.98	31
H3	1085.79	433.58	3597.47	35
H4	979.23	1089.99	4844.24	33
H5	3735.5	3370.52	6804.86	38
H6	6169.7	4551.86	7146.17	37
H7	8874.6	4802.76	6274.51	36
H8	9135.74	3845.8	5057.5	34
H11	7590(90)	2000(90)	3060(40)	20(17)
H12	6209.44	490.85	2003.59	33
H16A	10850.19	-3232.78	2196.82	61
H16B	9732.93	-2044.37	2836.8	61
H16C	11578.5	-1422.48	2324.1	61
H18A	10365.84	1957.12	-249.31	80
H18B	11224.32	2590.91	418.57	80
H18C	9265.73	3352.81	301.41	80
H20	4203.17	2189.25	1367.98	38
H21	3180.4	4807.56	830.83	46
H23	7190.69	7188.35	1444.64	44
H24	8186.2	4576.06	2001.66	39

Table S8. Crystal data and structure refinement for 4a.

Identification code	V3
Empirical formula	$C_{26}H_{21}BrNO_4P$
Formula weight	522.32
Temperature/K	160.0(2)
Crystal system	monoclinic
Space group	$P2_1/c$
a/Å	10.8242(3)
b/Å	8.2331(3)
c/Å	24.9042(5)
α/°	90
β/°	93.006(2)
γ/°	90
Volume/Å^3	2216.33(11)
Z	4
ρ_{calc}g/cm^3	1.565
μ/mm^{-1}	3.505
F(000)	1064.0
Crystal size/mm^3	0.12 × 0.11 × 0.01
Radiation	CuKα (λ = 1.54184 Å)
2Θ max. for data collection/°	160
Index ranges	$-13 \leq h \leq 13$, $-10 \leq k \leq 10$, $-21 \leq l \leq 31$
Reflections collected	22314
Independent reflections	4782 [R_{int} = 0.0738, R_{sigma} = 0.0629]
Data/restraints/parameters	4782/0/341
Goodness-of-fit on F^2	1.042
Final R indexes [$I > 2\sigma(I)$]	R_1 = 0.0541, wR_2 = 0.1388
Final R indexes [all data]	R_1 = 0.0634, wR_2 = 0.1480
Largest diff. peak/hole / e Å^{-3}	0.81/-0.45

Table S9. Fractional Atomic Coordinates (×10[4]) and Equivalent Isotropic Displacement Parameters (Å[2]×10[3]) for 4a. U_{eq} is defined as 1/3 of of the trace of the orthogonalised U_{IJ} tensor.

**Atom**	***x***	***y***	***z***	***U*(eq)**
C1	954(2)	3989(4)	2329.5(10)	34.6(5)
C2	1570(2)	3799(4)	2836.1(10)	35.1(5)
C3	2124(2)	2349(4)	2983.9(10)	34.3(5)
C4	2502(3)	-548(4)	2746.3(11)	38.3(6)
C5	2421(3)	-1815(4)	2389.5(12)	42.8(6)
C6	1820(3)	-1609(4)	1885.8(12)	43.0(6)
C7	618(3)	34(4)	1210.6(11)	43.3(6)
C8	-377(3)	1880(5)	533.1(11)	48.2(8)
C9	-772(3)	3383(5)	364.8(11)	51.0(8)
C10	-643(3)	4704(5)	711.7(13)	50.7(8)
C11	-135(3)	4508(4)	1227.7(12)	44.0(7)
C12	283(2)	2976(4)	1412.4(10)	36.0(6)
C13	160(3)	1654(4)	1058.5(10)	39.9(6)
C14	1297(3)	-125(4)	1742.7(11)	37.1(6)
C15	1382(2)	1222(3)	2102.0(10)	32.6(5)
C16	860(2)	2763(3)	1954.9(10)	32.3(5)
C17	2009(2)	996(3)	2617.8(10)	32.2(5)
N18	2817(2)	2169(3)	3462.1(9)	39.3(5)
C19	2950(3)	3445(4)	3860.0(10)	38.0(6)
C20	3662(2)	4926(4)	3686.4(9)	33.9(5)
C21	4530(3)	4823(4)	3292.0(11)	38.7(6)
C22	5222(3)	6162(4)	3167.0(11)	42.0(6)
C23	5063(3)	7590(4)	3443.9(11)	38.3(6)
C24	4218(3)	7730(4)	3840.2(11)	37.9(6)
C25	3507(3)	6388(4)	3948.8(10)	37.5(6)
Br26	6014.5(4)	9450.8(5)	3272.0(2)	56.81(16)
P27	3890(2)	2509(4)	4437.7(11)	35.0(5)
O29	5246(3)	2719(5)	4263.6(13)	43.4(7)
C30	6250(8)	2035(10)	4578(3)	52.6(17)
O31	3798(3)	3742(4)	4910.4(12)	43.2(7)
C32	2898(10)	3533(14)	5325(4)	66(2)
O28	3431(2)	869(3)	4545.8(8)	48.2(5)
P27A	3487(4)	2663(7)	4496(2)	37.6(9)
O29A	4862(6)	3230(8)	4588(3)	50.4(16)
C30A	5908(19)	2260(30)	4527(10)	75(6)
O31A	2795(7)	3735(9)	4898(2)	50.2(15)
C32A	3130(20)	3580(30)	5465(9)	66(6)
O33	465(3)	-1154(3)	913.2(10)	66.1(7)

Table S10 Anisotropic Displacement Parameters ($Å^2×10^3$) for 4a. The Anisotropic displacement factor exponent takes the form: $-2\pi^2[h^2a^{*2}U_{11}+2hka^*b^*U_{12}+…]$.

Atom	U_{11}	U_{22}	U_{33}	U_{23}	U_{13}	U_{12}
C1	33.9(12)	40.9(14)	28.5(11)	0.8(10)	-2.1(9)	4.3(10)
C2	34.9(12)	43.2(14)	26.9(11)	-3.7(10)	-2.3(9)	2.9(11)
C3	35.3(12)	42.6(14)	24.6(11)	1.2(10)	-2.2(9)	-4.1(10)
C4	41.7(14)	41.9(15)	30.8(12)	5.0(11)	-3.8(10)	-0.7(11)
C5	50.5(16)	33.2(14)	44.5(15)	0.4(12)	-0.2(12)	-1.3(12)
C6	52.9(16)	36.3(14)	39.7(14)	-5.5(12)	1.6(12)	-7.4(12)
C7	49.6(16)	49.3(17)	30.5(12)	-5.5(12)	-1.7(11)	-11.2(13)
C8	42.8(14)	74(2)	27.2(12)	-3.3(13)	-2.9(11)	-11.8(14)
C9	39.0(14)	85(3)	28.5(13)	9.5(15)	-5.5(10)	-7.6(15)
C10	43.5(15)	70(2)	37.5(15)	13.5(15)	-2.6(12)	3.4(15)
C11	44.4(15)	56.9(19)	30.1(13)	4.2(12)	-2.9(11)	3.6(13)
C12	31.5(11)	48.8(16)	27.6(11)	3.0(11)	0.6(9)	-1.9(11)
C13	38.4(13)	54.1(17)	26.8(12)	-0.3(11)	-2.0(10)	-7.6(12)
C14	40.3(13)	40.4(14)	30.8(12)	-3.2(11)	2.0(10)	-7.1(11)
C15	31.7(11)	39.3(14)	26.8(11)	0.4(10)	1.5(9)	-4.4(10)
C16	29.7(11)	41.5(14)	25.6(11)	0.7(10)	-0.3(9)	-1.3(10)
C17	33.0(11)	35.8(13)	27.8(11)	1.3(10)	1.0(9)	-3.0(10)
N18	49.4(13)	36.5(13)	30.6(11)	1.3(10)	-10.7(9)	-1.6(10)
C19	44.4(14)	43.7(15)	24.9(11)	-0.5(10)	-6.6(10)	-1.7(12)
C20	36.4(12)	40.6(14)	24.0(11)	0.4(10)	-5.7(9)	2.7(11)
C21	40.6(13)	44.3(15)	30.9(12)	-9.0(11)	-0.7(10)	7.2(11)
C22	37.5(13)	52.7(17)	35.9(13)	-6.3(12)	5.1(10)	1.5(12)
C23	38.7(13)	45.0(15)	31.0(12)	1.0(11)	-1.6(10)	0.6(11)
C24	45.4(14)	37.4(14)	30.8(12)	0.0(11)	0.5(10)	3.3(11)
C25	40.4(13)	45.1(15)	27.0(11)	-0.9(11)	2.6(10)	3.7(11)
Br26	61.2(2)	56.2(2)	53.9(2)	3.80(15)	10.93(16)	-13.20(16)
P27	42.0(12)	37.5(8)	24.4(7)	-1.4(5)	-8.5(8)	-0.1(9)
O29	44.5(17)	56(2)	29.4(14)	8.9(15)	-4.4(13)	6.7(15)
C30	56(4)	62(4)	39(3)	5(2)	-17(3)	9(3)
O31	56(2)	45.1(18)	28.4(14)	-2.2(13)	-2.8(13)	-5.6(15)
C32	62(5)	85(6)	53(6)	-1(4)	18(4)	13(4)
O28	63.3(13)	42.9(12)	37.0(10)	1.9(9)	-8.7(9)	-5.5(10)
P27A	41(2)	40.3(17)	30.3(17)	2.2(12)	-12.6(16)	1.2(17)
O29A	43(3)	45(3)	61(4)	9(3)	-19(3)	-11(3)
O31A	58(4)	62(4)	29(3)	2(3)	-9(3)	9(3)
O33	101(2)	54.6(15)	40.7(12)	-14.2(11)	-15.1(12)	-10.9(14)

Table S11 Bond Lengths for 4a.

Atom	Atom	Length/Å	Atom	Atom	Length/Å
C1	C2	1.404(3)	N18	C19	1.447(4)
C1	C16	1.375(4)	C19	C20	1.517(4)
C2	C3	1.377(4)	C19	P27	1.883(4)
C3	C17	1.441(4)	C19	P27A	1.778(6)
C3	N18	1.382(3)	C20	C21	1.397(4)
C4	C5	1.370(4)	C20	C25	1.384(4)
C4	C17	1.408(4)	C21	C22	1.378(5)
C5	C6	1.393(4)	C22	C23	1.378(4)
C6	C14	1.385(4)	C23	C24	1.385(4)
C7	C13	1.465(5)	C23	Br26	1.907(3)
C7	C14	1.487(4)	C24	C25	1.381(4)
C7	O33	1.233(4)	P27	O29	1.562(4)
C8	C9	1.368(6)	P27	O31	1.561(4)
C8	C13	1.416(4)	P27	O28	1.468(4)
C9	C10	1.391(6)	O29	C30	1.422(7)
C10	C11	1.381(4)	O31	C32	1.465(9)
C11	C12	1.409(4)	O28	P27A	1.484(6)
C12	C13	1.403(4)	P27A	O29A	1.565(7)
C12	C16	1.469(3)	P27A	O31A	1.557(8)
C14	C15	1.425(4)	O29A	C30A	1.40(2)
C15	C16	1.429(4)	O31A	C32A	1.44(2)
C15	C17	1.433(3)			

Table S12 Bond Angles for 4a.

Atom	Atom	Atom	Angle/°	Atom	Atom	Atom	Angle/°
C16	C1	C2	122.9(3)	C3	N18	C19	122.8(3)
C3	C2	C1	121.1(3)	N18	C19	C20	115.0(2)
C2	C3	C17	118.7(2)	N18	C19	P27	104.9(2)
C2	C3	N18	122.2(3)	N18	C19	P27A	111.3(3)
N18	C3	C17	119.1(3)	C20	C19	P27	106.5(2)
C5	C4	C17	121.9(3)	C20	C19	P27A	113.4(3)
C4	C5	C6	120.2(3)	C21	C20	C19	121.5(3)
C14	C6	C5	120.3(3)	C25	C20	C19	119.2(2)
C13	C7	C14	117.1(3)	C25	C20	C21	119.2(3)
O33	C7	C13	122.2(3)	C22	C21	C20	120.3(3)
O33	C7	C14	120.6(3)	C23	C22	C21	119.0(2)
C9	C8	C13	120.6(3)	C22	C23	C24	122.1(3)
C8	C9	C10	119.8(3)	C22	C23	Br26	119.3(2)
C11	C10	C9	120.6(3)	C24	C23	Br26	118.5(2)
C10	C11	C12	120.9(3)	C25	C24	C23	118.0(3)
C11	C12	C16	121.4(3)	C24	C25	C20	121.4(2)
C13	C12	C11	118.1(2)	O29	P27	C19	102.7(2)
C13	C12	C16	120.5(3)	O31	P27	C19	104.9(2)
C8	C13	C7	118.4(3)	O31	P27	O29	103.6(2)
C12	C13	C7	121.6(2)	O28	P27	C19	110.0(2)
C12	C13	C8	120.0(3)	O28	P27	O29	119.0(2)
C6	C14	C7	118.9(3)	O28	P27	O31	115.1(2)
C6	C14	C15	120.8(3)	C30	O29	P27	120.4(4)
C15	C14	C7	120.2(3)	C32	O31	P27	121.7(5)
C14	C15	C16	121.2(2)	O28	P27A	C19	115.0(4)
C14	C15	C17	118.3(3)	O28	P27A	O29A	109.1(4)
C16	C15	C17	120.5(2)	O28	P27A	O31A	119.1(5)
C1	C16	C12	123.3(3)	O29A	P27A	C19	106.7(4)
C1	C16	C15	117.6(2)	O31A	P27A	C19	102.8(4)
C15	C16	C12	119.0(2)	O31A	P27A	O29A	102.8(5)
C4	C17	C3	122.4(2)	C30A	O29A	P27A	125.5(11)
C4	C17	C15	118.5(2)	C32A	O31A	P27A	118.2(11)
C15	C17	C3	119.2(2)				

Table S13. Hydrogen Atom Coordinates (Å×10^4) and Isotropic Displacement Parameters (Å^2×10^3) for 4a.

**Atom**	***x***	***y***	***z***	***U*(iso)**
H1	587.3	5010.01	2241.77	41
H2	1605.33	4685.3	3080.83	42
H4	2901.62	-715.69	3090.47	46
H5	2775.02	-2836.24	2485.98	51
H6	1769.38	-2489.43	1639.22	52
H8	-463.25	979.94	295.29	58
H9	-1134.46	3525.06	12.1	61
H10	-906.36	5750.2	592.36	61
H11	-66.26	5419.05	1461.58	53
H18	2880(30)	1340(50)	3571(15)	35(9)
H19	2110(40)	3820(50)	3983(16)	50(10)
H21	4642.67	3825.04	3109.24	46
H22	5801.2	6102.03	2893.83	50
H24	4130.41	8717.81	4031.5	45
H25	2898.36	6470.22	4208.91	45
H30A	7028.17	2282.71	4410.85	79
H30B	6269.73	2495.39	4940.94	79
H30C	6144.52	854.57	4597.89	79
H32A	2984.28	4424.82	5584.47	99
H32B	2059.46	3536.7	5156.46	99
H32C	3050.15	2496.48	5510.36	99
H30D	6656.91	2898.16	4608.46	113
H30E	5889.16	1331.01	4773.91	113
H30F	5908.42	1859.72	4156.04	113
H32D	2625.05	4318.28	5669.47	99
H32E	2988.09	2457.52	5579.31	99
H32F	4007.05	3850.53	5530.05	99

Table S14. Atomic Occupancy for 4a.

**Atom**	***Occupancy***	**Atom**	***Occupancy***	**Atom**	***Occupancy***
P27	0.65	O29	0.65	C30	0.65
H30A	0.65	H30B	0.65	H30C	0.65
O31	0.65	C32	0.65	H32A	0.65
H32B	0.65	H32C	0.65	P27A	0.35
O29A	0.35	C30A	0.35	H30D	0.35
H30E	0.35	H30F	0.35	O31A	0.35
C32A	0.35	H32D	0.35	H32E	0.35
H32F	0.35				

Experimental

Single crystals of $C_{23}H_{19}BrNO_5P$ [2a] were investigated on a Rigaku, XtaLAB Synergy, Dualflex, HyPix diffractometer. The crystal was kept at 150.0(3) K during data collection. Using Olex2 [1], the structure was solved with the ShelXS [2] structure solution program using Patterson Method and refined with the ShelXL [3] refinement package using Least Squares minimisation.

1. Dolomanov, O.V., Bourhis, L.J., Gildea, R.J, Howard, J.A.K. & Puschmann, H. (2009), J. Appl. Cryst. 42, 339-341.
2. Sheldrick, G.M. (2008). Acta Cryst. A64, 112-122.
3. Sheldrick, G.M. (2015). Acta Cryst. C71, 3-8.

Crystal Data [2a] for $C_{23}H_{19}BrNO_5P$ (M = 500.27 g/mol): triclinic, space group $P\bar{1}$ (no. 2), a = 7.5626(2) Å, b = 7.7876(2) Å, c = 18.0152(5) Å, α = 85.582(2)°, β = 78.626(2)°, γ = 85.643(2)°, V = 1035.07(5) Å^3, Z = 2, T = 150.0(3) K, μ(CuKα) = 3.759 mm^{-1}, $Dcalc$ = 1.605 g/cm^3, 17799 reflections measured (2Θ ≤ 160°), 4456 unique (R_{int} = 0.0343, R_{sigma} = 0.0246) which were used in all calculations. The final R_1 was 0.0770 ($I > 2\sigma(I)$) and wR_2 was 0.2016 (all data).

Details:

```
1. Fixed Uiso
 At 1.2 times of:
  All C(H) groups
 At 1.5 times of:
  All C(H,H,H) groups
2.a Ternary CH refined with riding coordinates:
 C12(H12)
2.b Aromatic/amide H refined with riding coordinates:
 C2(H2), C3(H3), C4(H4), C5(H5), C6(H6), C7(H7), C8(H8), C20(H20), C21(H21),
 C23(H23), C24(H24)
2.c Idealised Me refined as rotating group:
 C16(H16A,H16B,H16C), C18(H18A,H18B,H18C)
```

This report has been created with Olex2, compiled on 2018.05.17 svn.r3504 for Rigaku Oxford Diffraction.

Single crystals of $C_{26}H_{21}BrNO_4P$ [4a] were investigated on a Rigaku, XtaLAB Synergy, Dualflex, HyPix diffractometer. The crystal was kept at 160.0(2) K during data collection. Using Olex2 [1], the structure was solved with the SIR2011 [2] structure solution program using Direct Methods and refined with the ShelXL [3] refinement package using Least Squares minimisation.

1. Dolomanov, O.V., Bourhis, L.J., Gildea, R.J, Howard, J.A.K. & Puschmann, H. (2009), J. Appl. Cryst. 42, 339-341.
2. Burla, M.C., Caliandro, R., Camalli, M., Carrozzini, B., Cascarano, G.L., De Caro, L., Giacovazzo, C., Polidori, G., Siliqi, D., Spagna, R. (2007). J. Appl. Cryst. 40, 609-613.
3. Sheldrick, G.M. (2015). Acta Cryst. C71, 3-8.

Crystal Data [4a] for $C_{26}H_{21}BrNO_4P$ (M = 522.32 g/mol): monoclinic, space group $P2_1/c$ (no.14), a = 10.8242(3) Å, b = 8.2331(3) Å, c = 24.9042(5) Å, β = 93.006(2)°, V = 2216.3(1) Å^3, Z = 4, T = 160.0(2) K, μ(CuKα) = 3.450 mm^{-1}, $Dcalc$ = 1.517 g/cm^3, 22314 reflections measured (2Θ ≤ 160°), 4782 unique (R_{int} = 0.0738, R_{sigma} = 0.0629) which were used in all calculations. The final R_1 was 0.0541 ($I > 2\sigma(I)$) and wR_2 was 0.1480 (all data).

Details:

1. Fixed Uiso
 At 1.2 times of:
  All C(H) groups
 At 1.5 times of:
  All C(H,H,H) groups
2. Others
 Fixed Sof: P27(0.65) O29(0.65) C30(0.65) H30A(0.65) H30B(0.65) H30C(0.65)
 O31(0.65) C32(0.65) H32A(0.65) H32B(0.65) H32C(0.65) P27A(0.35) O29A(0.35)

C30A(0.35) H30D(0.35) H30E(0.35) H30F(0.35) O31A(0.35) C32A(0.35) H32D(0.35)
 H32E(0.35) H32F(0.35)
3.a Me refined with riding coordinates:
 C30(H30A,H30B,H30C), C32(H32A,H32B,H32C), C30A(H30D,H30E,H30F), C32A(H32D,
 H32E,H32F)
3.b Aromatic/amide H refined with riding coordinates:
 C1(H1), C2(H2), C4(H4), C5(H5), C6(H6), C8(H8), C9(H9), C10(H10), C11(H11),
 C21(H21), C22(H22), C24(H24), C25(H25)

This report has been created with Olex2, compiled on 2018.05.17 svn.r3504 for Rigaku Oxford Diffraction.

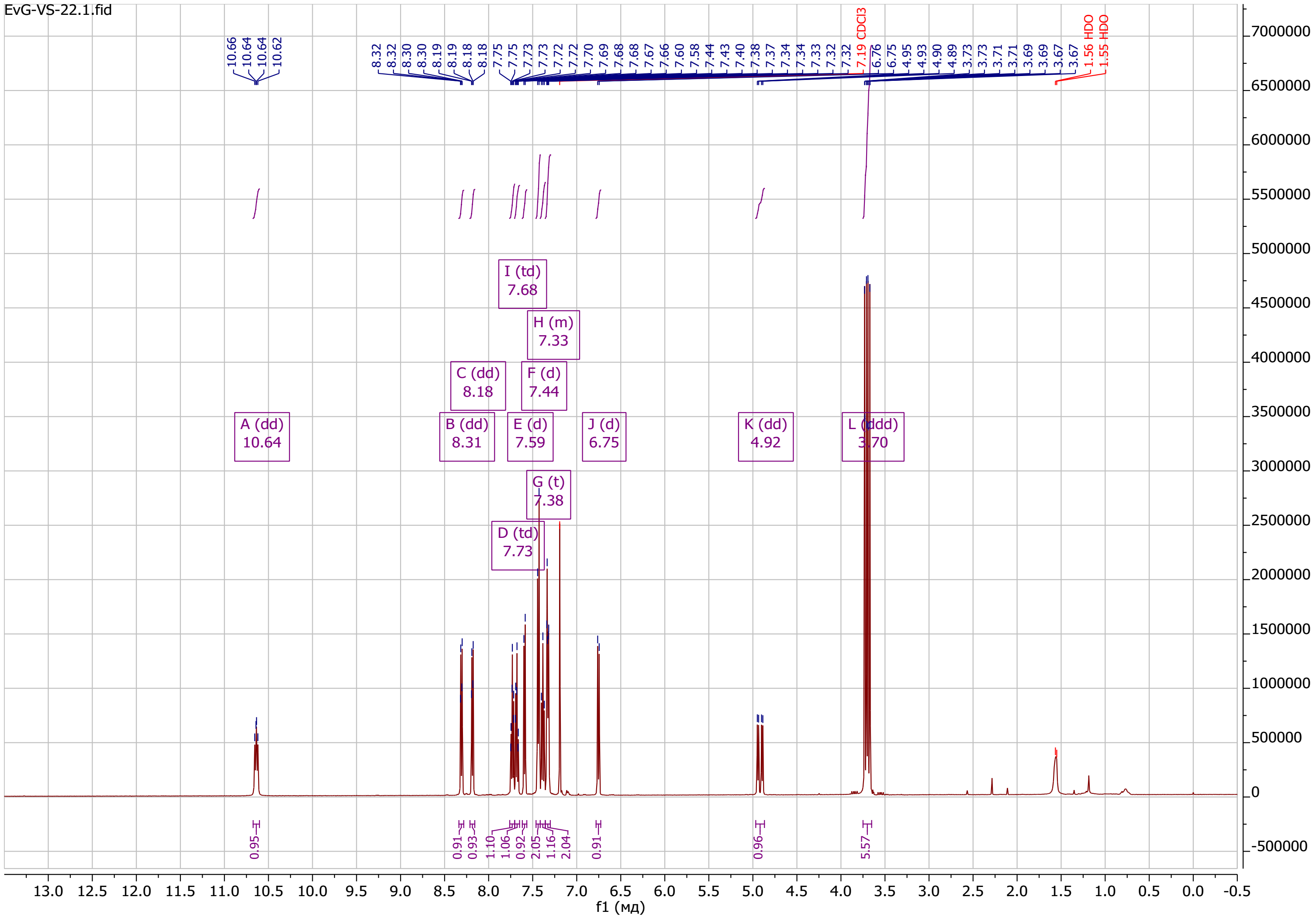

Figure S1. **2a** ^{1}H NMR (500 MHz, Chloroform-d) spectrum.

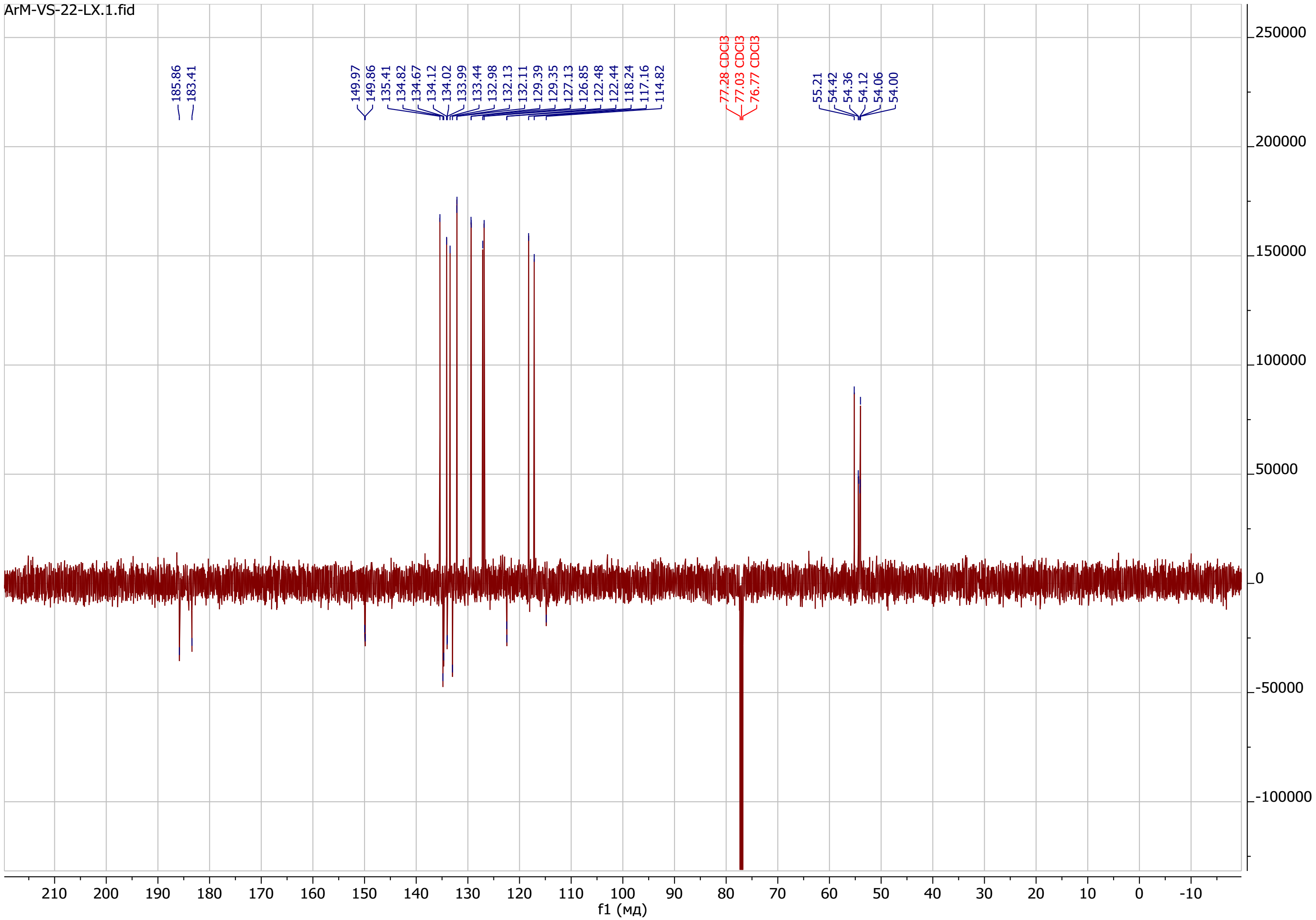

Figure S2. **2a** ^{13}C NMR (126 MHz, Chloroform-d) spectrum.

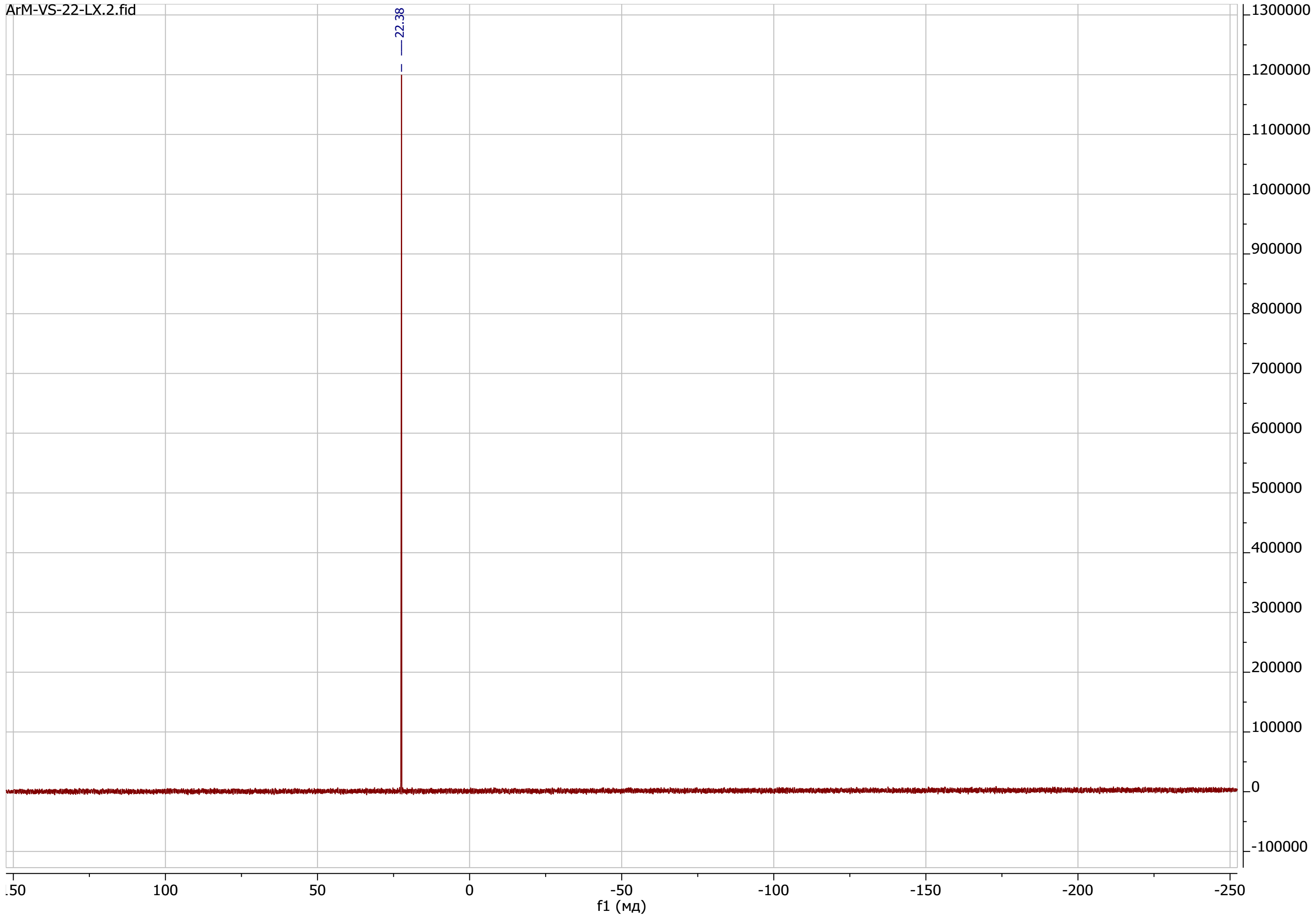

Figure S3. **2a** ^{31}P NMR (202 MHz, Chloroform-d) spectrum.

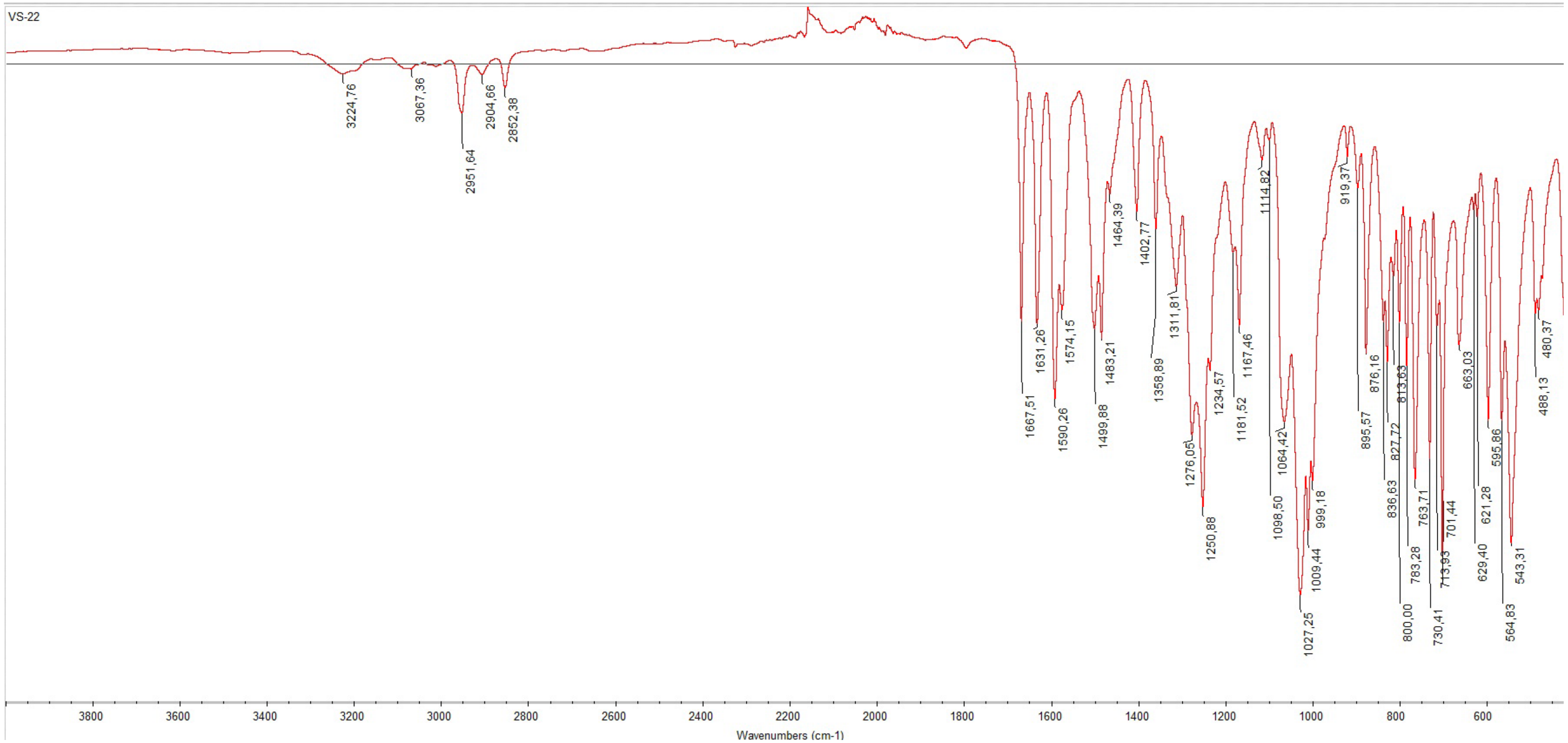

Figure S4. FTIR spectrum of **2a**.

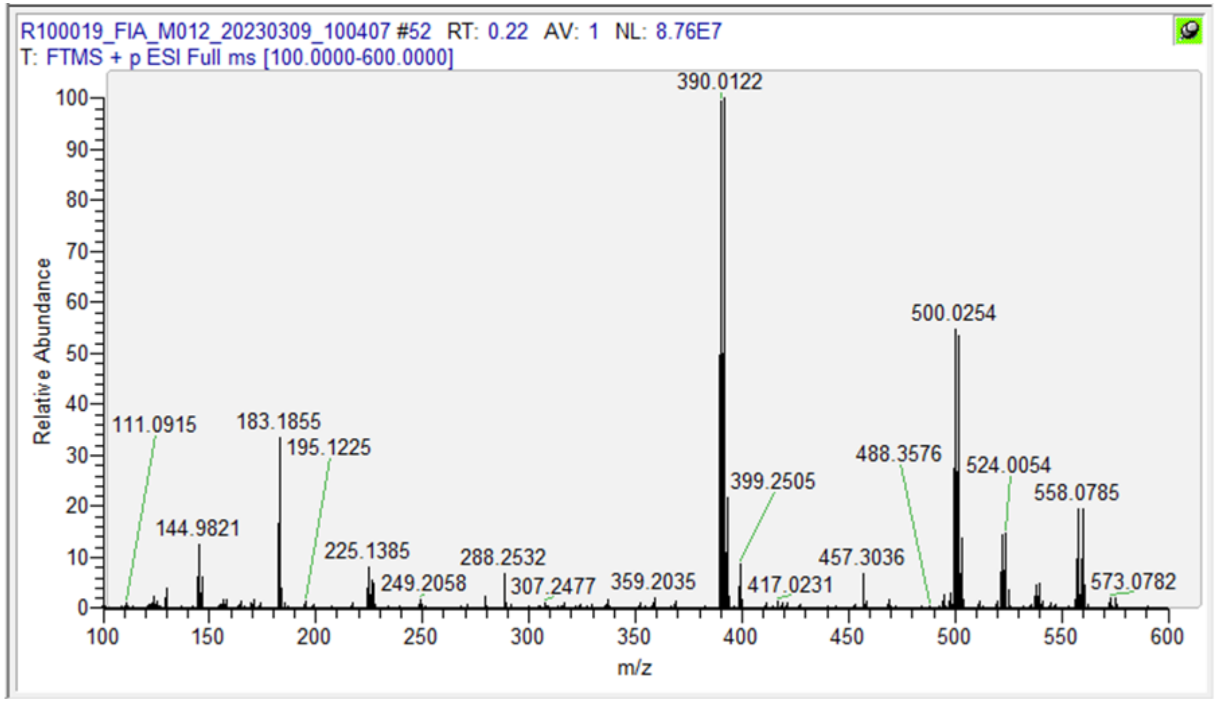

Figure S5. Mass spectrum of **2a** (ESI-FTMS).

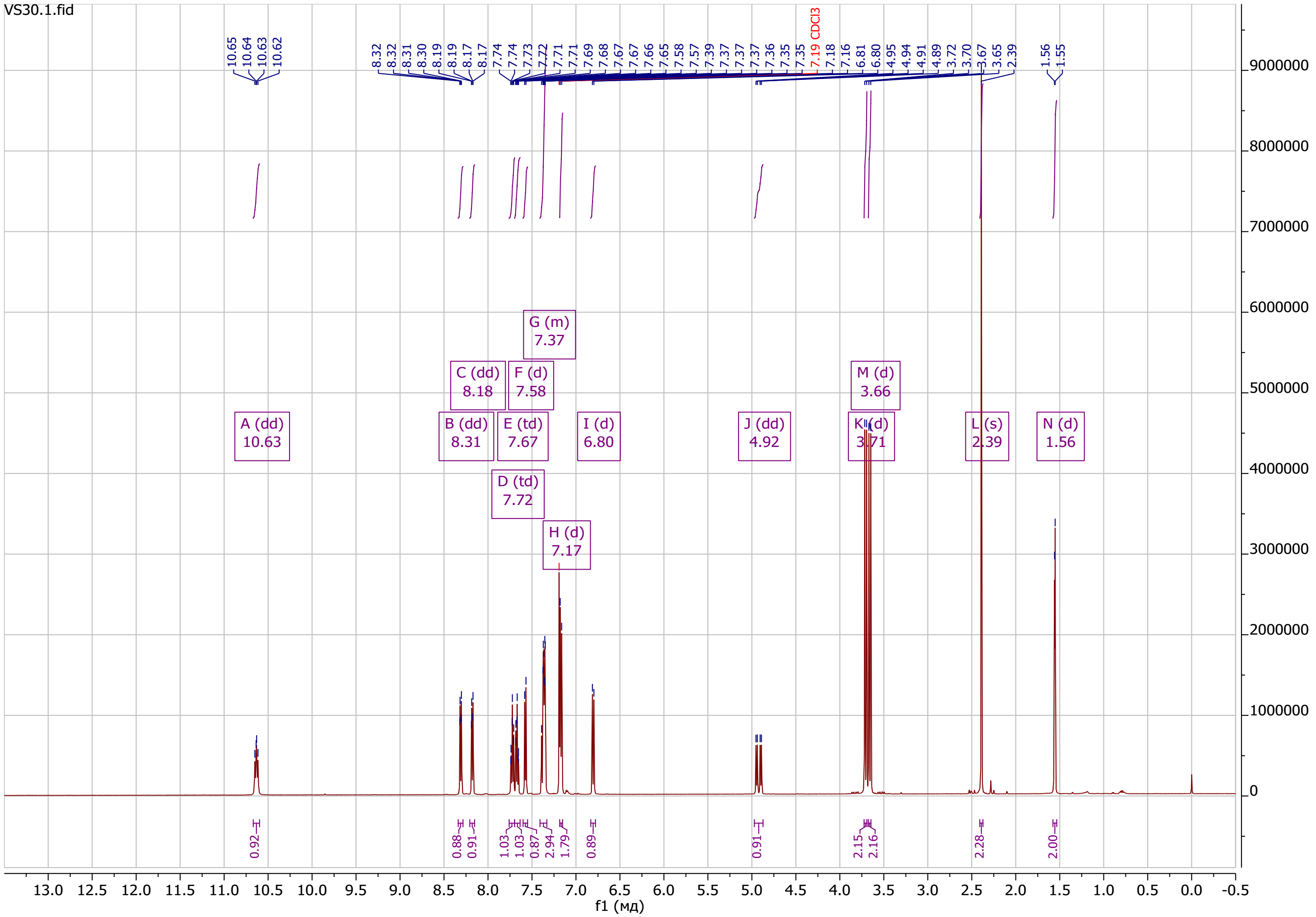

Figure S6. **2b** ^{1}H NMR (500 MHz, Chloroform-d) spectrum.

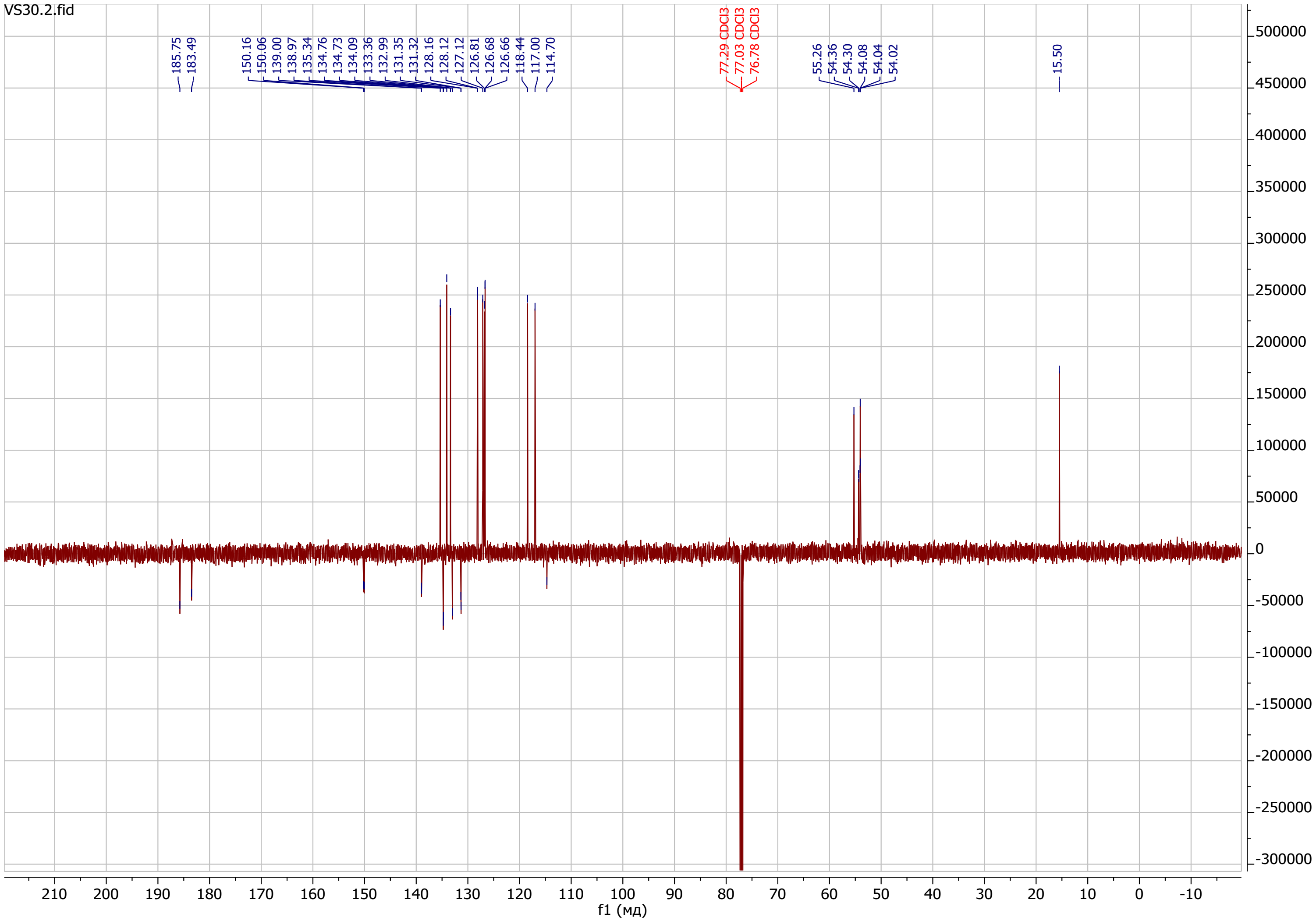

Figure S7. **2b** ^{13}C NMR (126 MHz, Chloroform-d) spectrum.

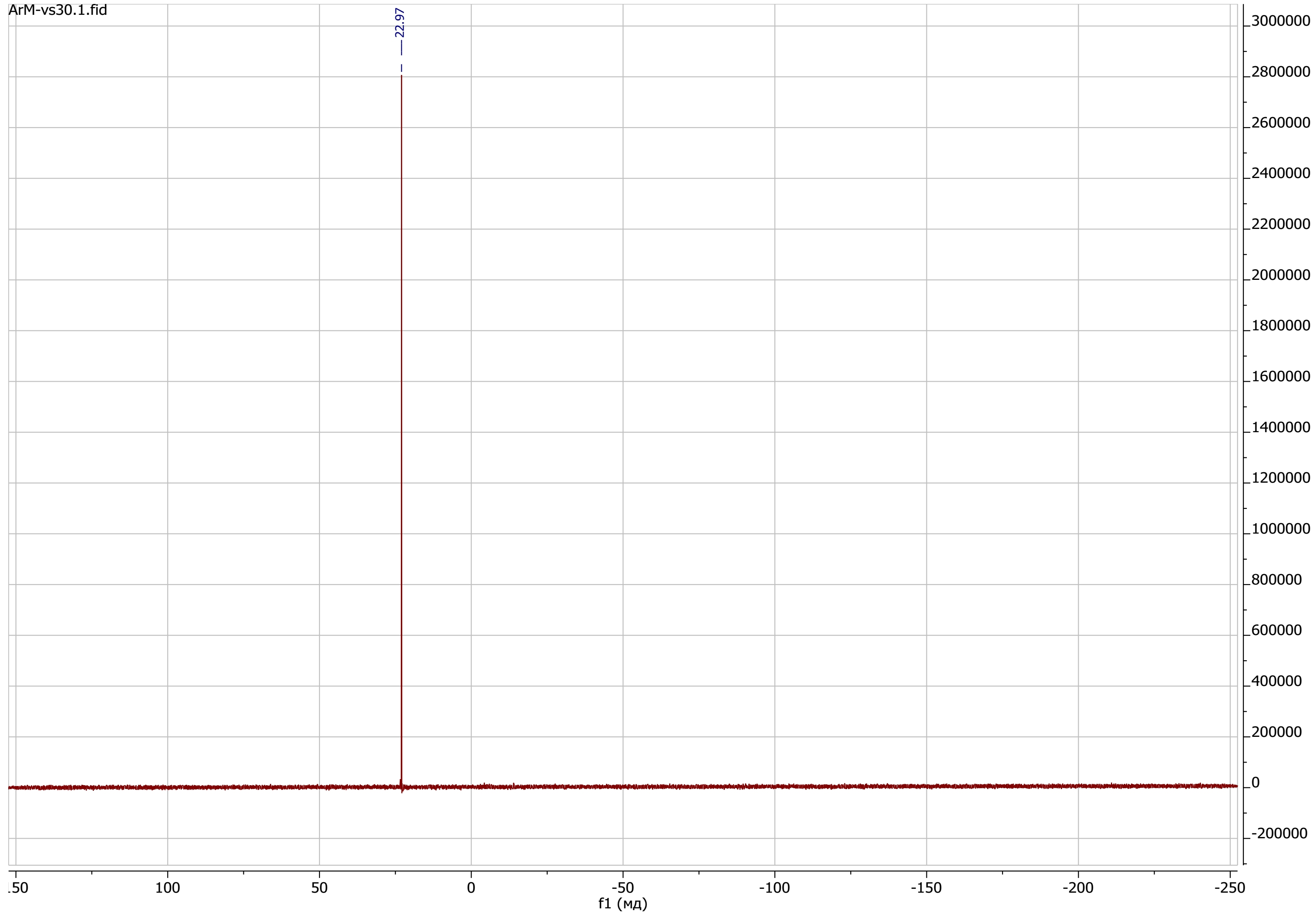

Figure S8. **2a** ^{31}P NMR (202 MHz, Chloroform-d) spectrum.

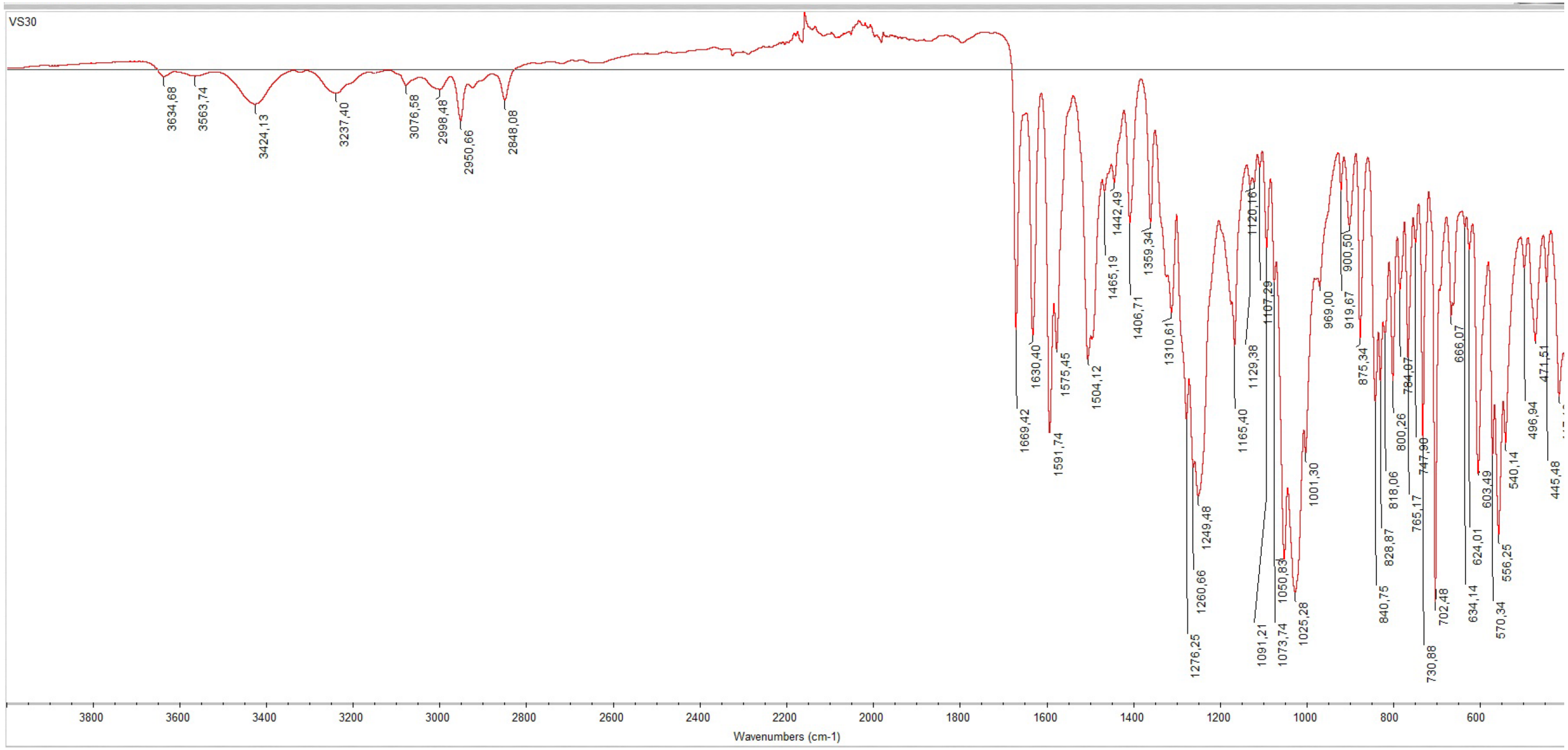

Figure S9. FTIR spectrum of **2b**.

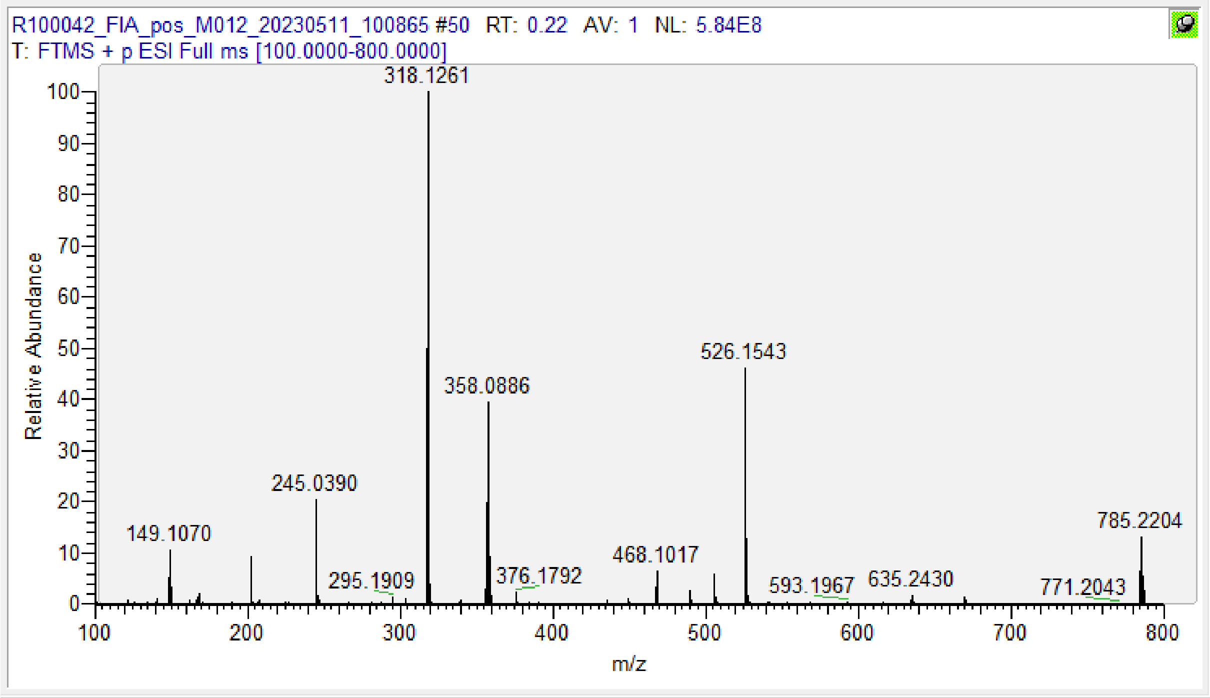

Figure S10. Mass spectrum of **2a** (ESI-FTMS).

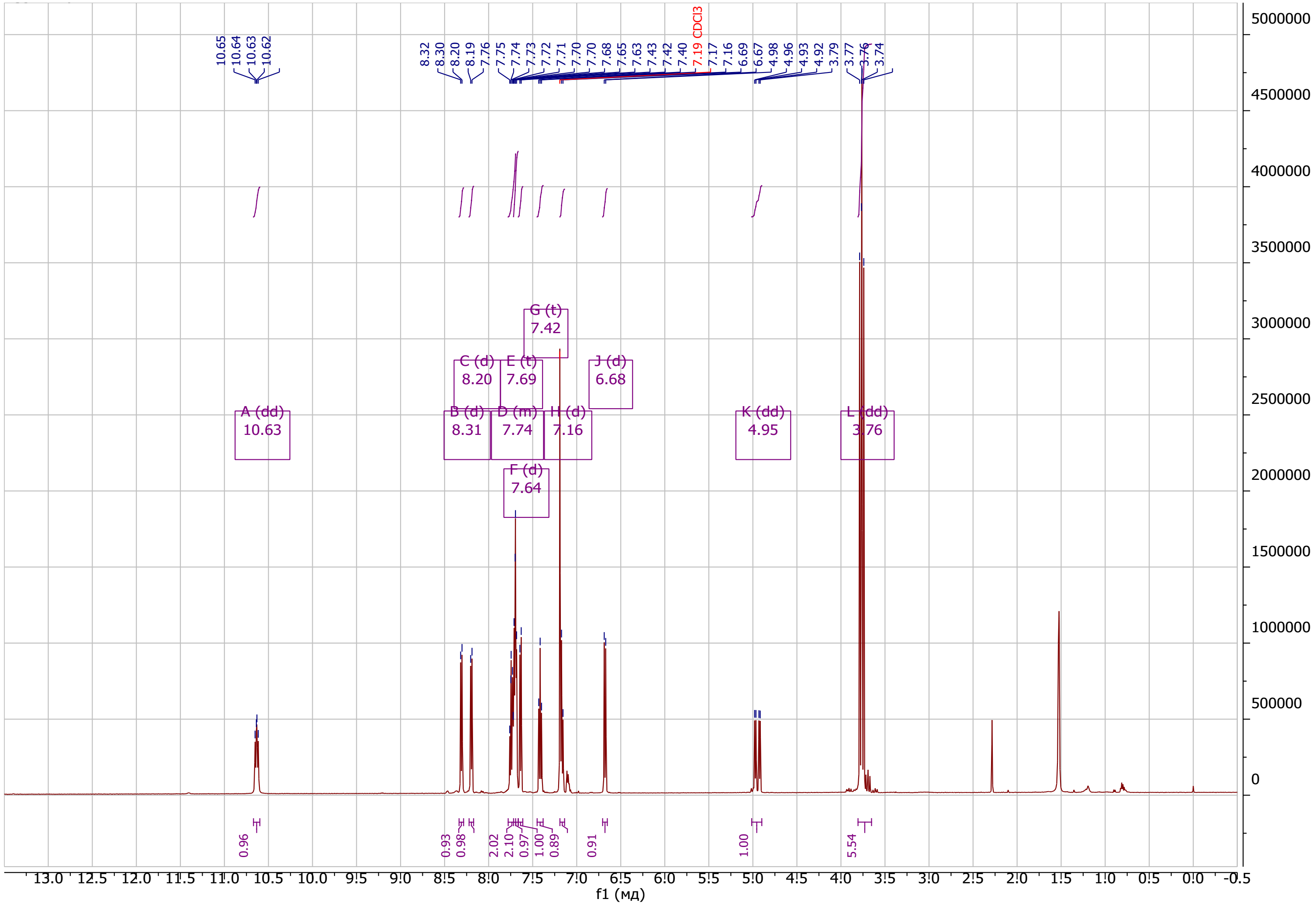

Figure S11. **2c** ^{1}H NMR (500 MHz, Chloroform-d) spectrum.

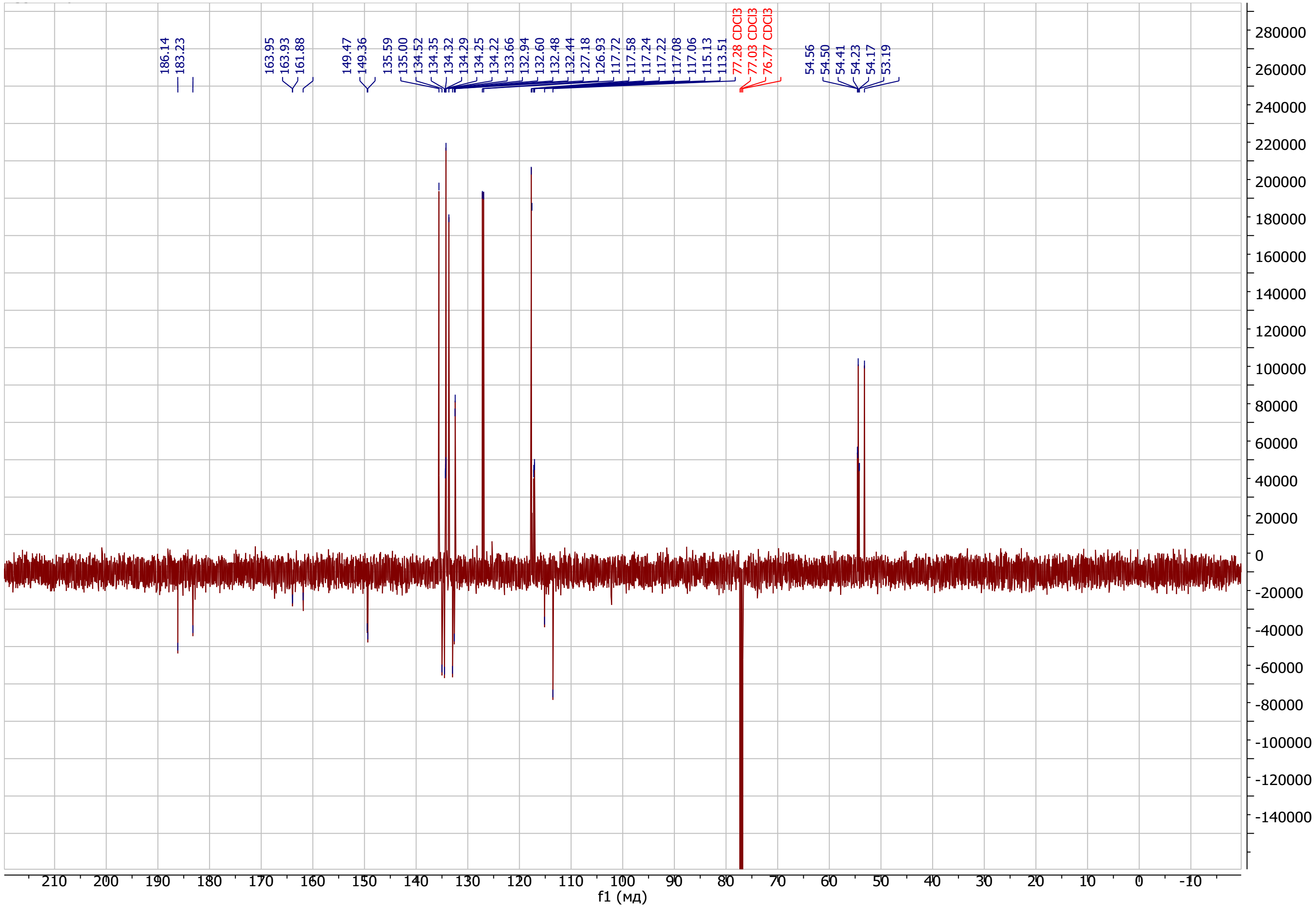

Figure S12. **2c** ^{13}C NMR (126 MHz, Chloroform-d) spectrum.

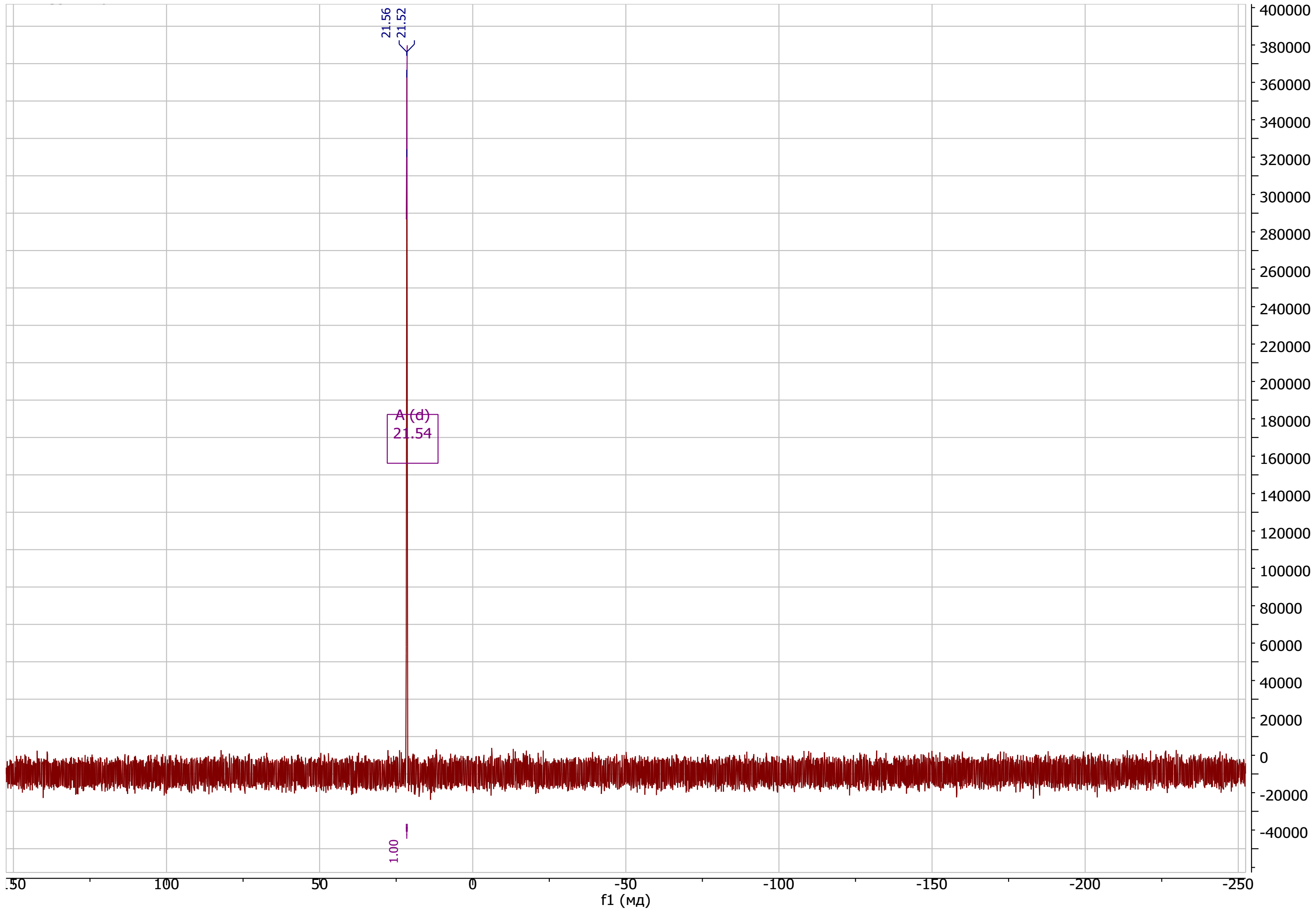

Figure S13. **2c** ^{31}P NMR (202 MHz, Chloroform-d) spectrum.

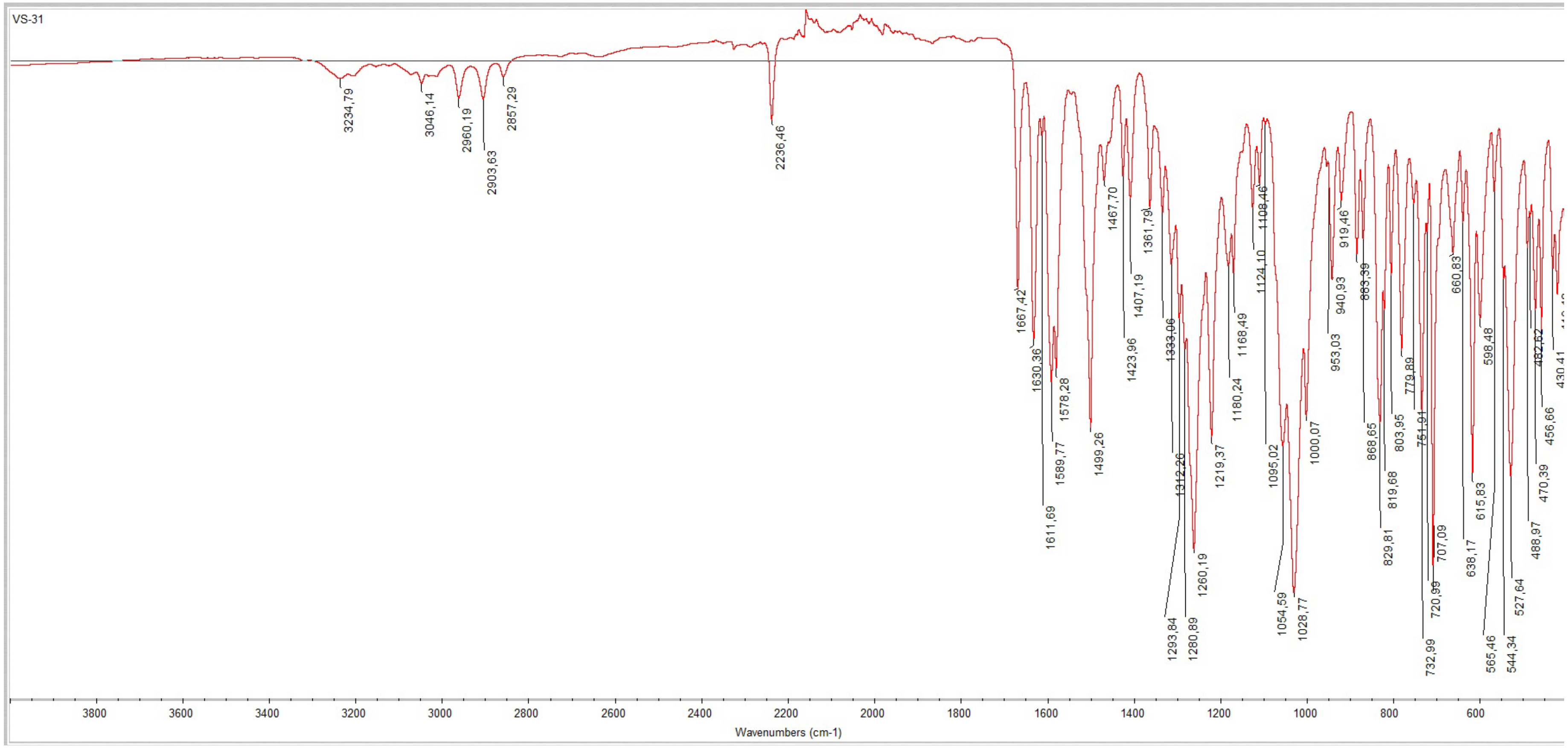

Figure S14. FTIR spectrum of **2c**.

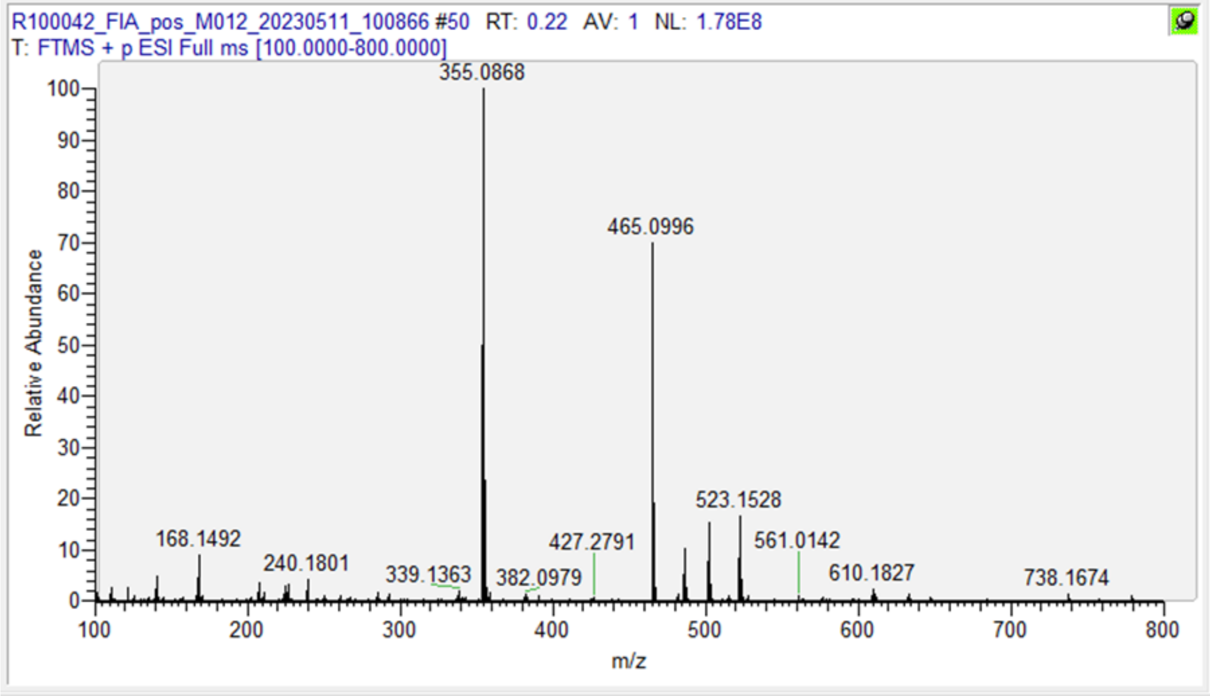

Figure S15. Mass spectrum of **2c** (ESI-FTMS).

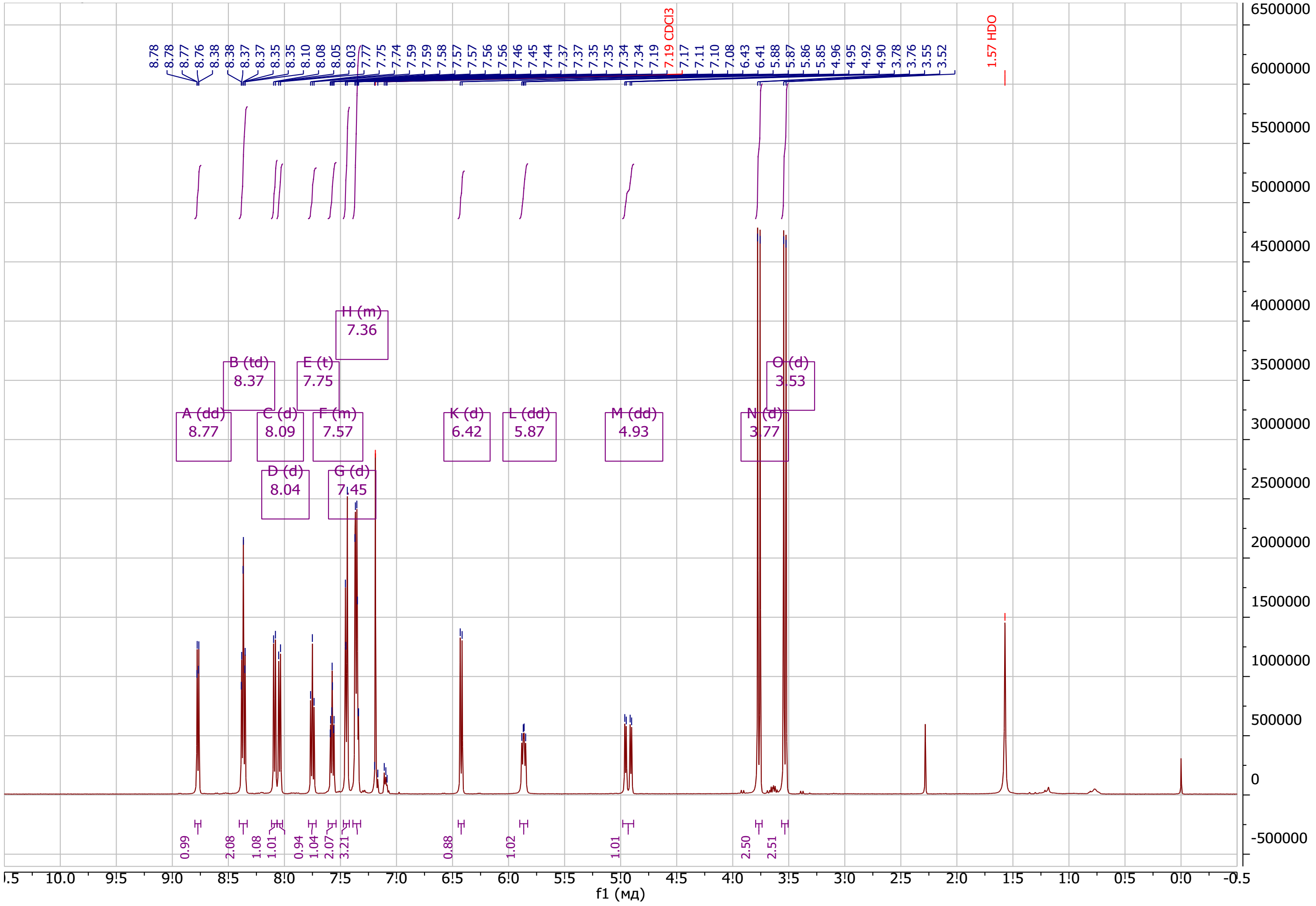

Figure S16. **4a** ^{1}H NMR (500 MHz, Chloroform-d) spectrum.

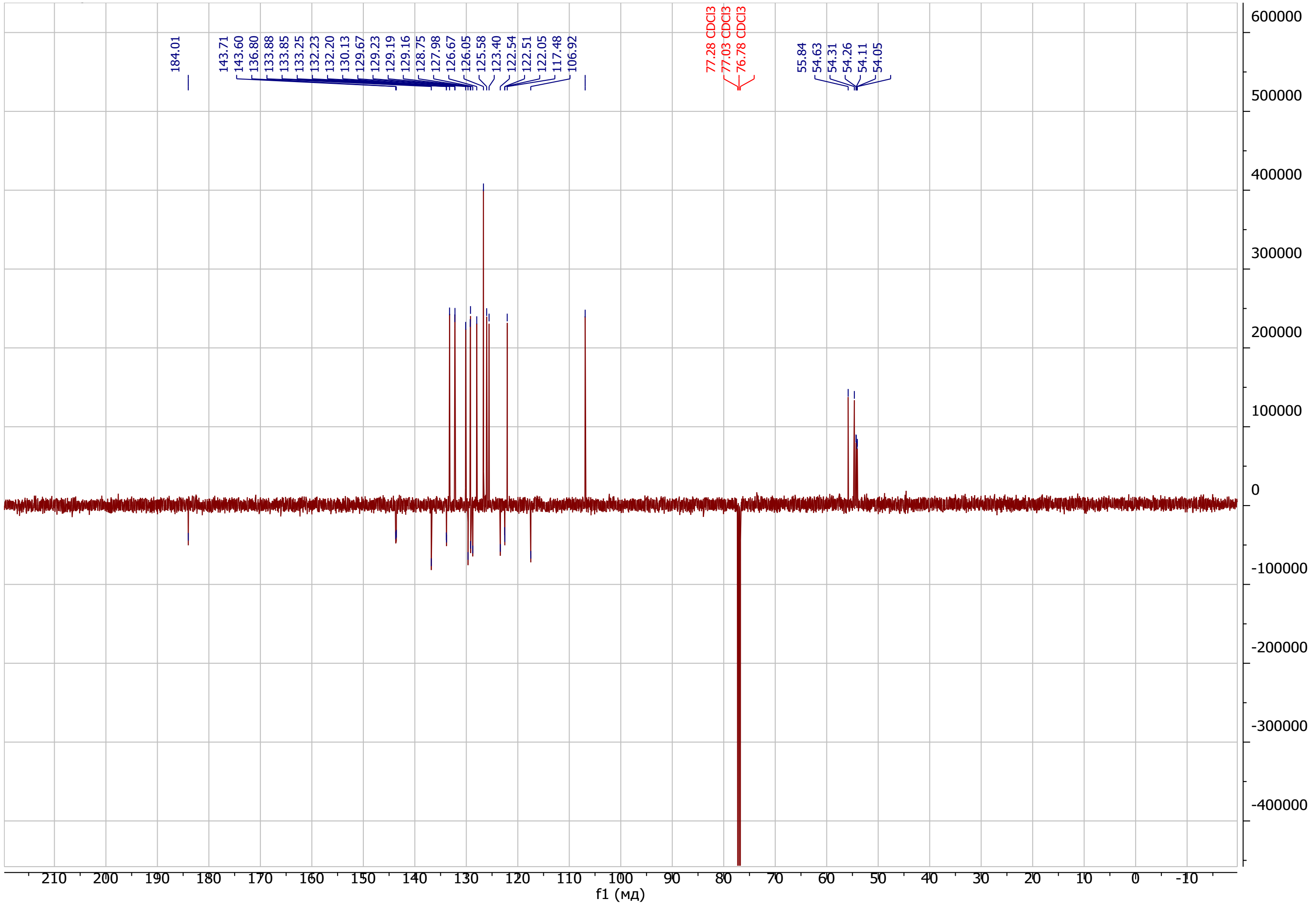

Figure S17. **4a** ^{13}C NMR (126 MHz, Chloroform-d) spectrum.

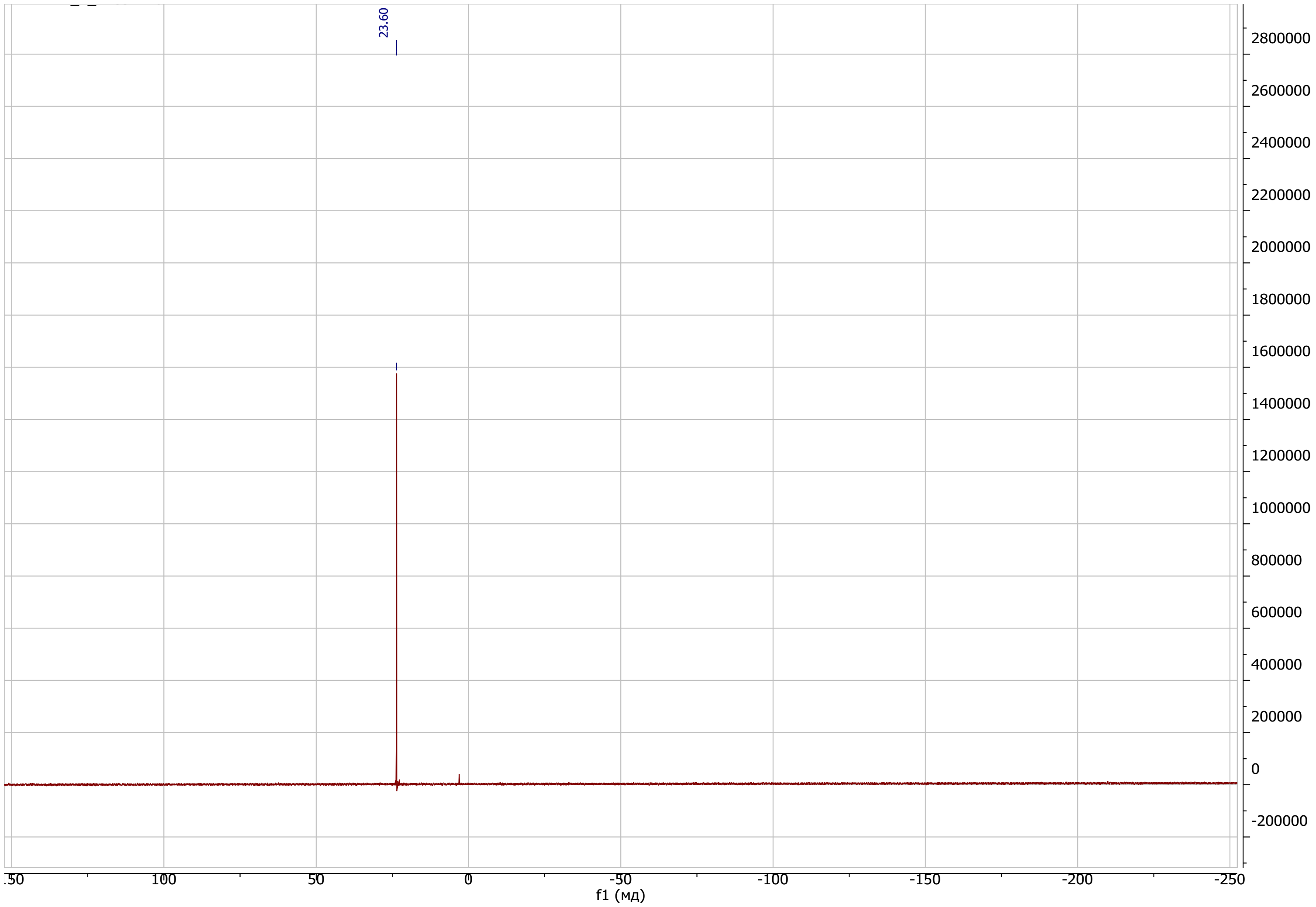

Figure S18. **4a** ^{31}P NMR (202 MHz, Chloroform-d) spectrum.

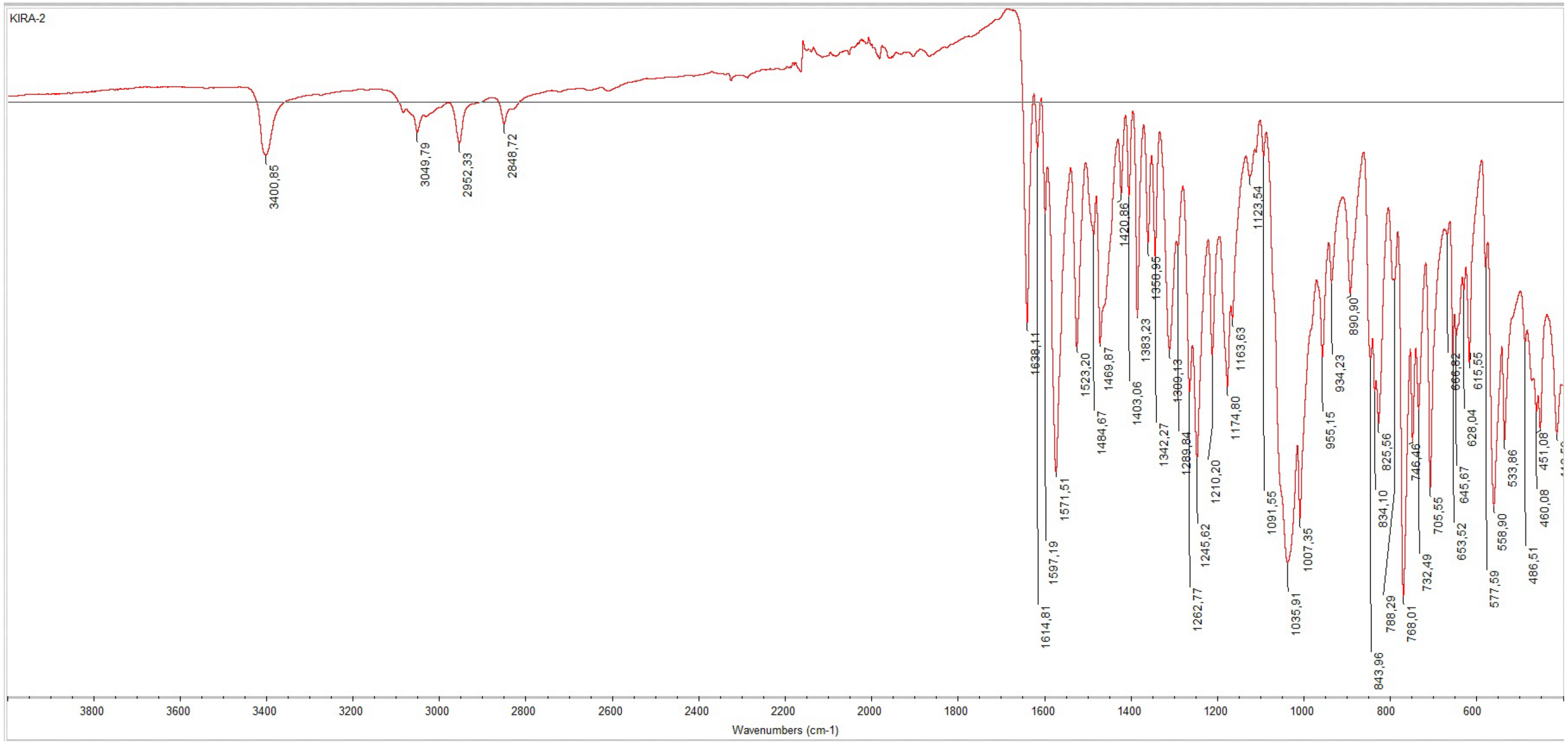

Figure S19. FTIR spectrum of **4a**.

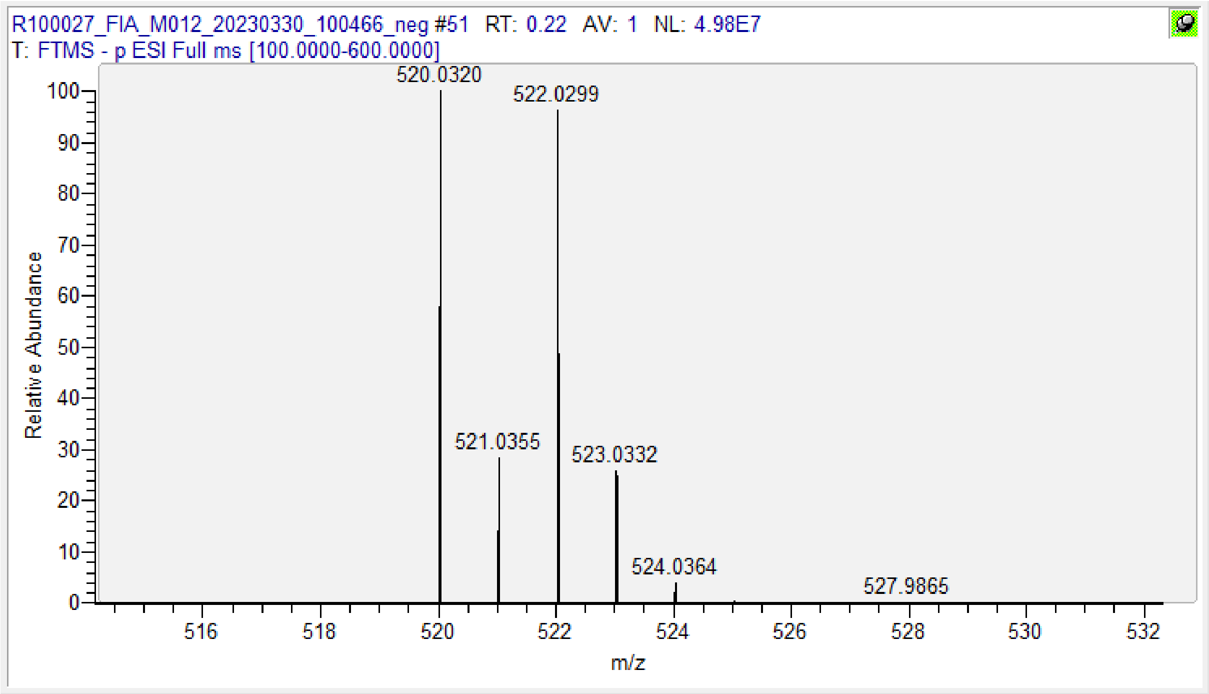

Figure S20. Mass spectrum of **4a** (ESI-FTMS).

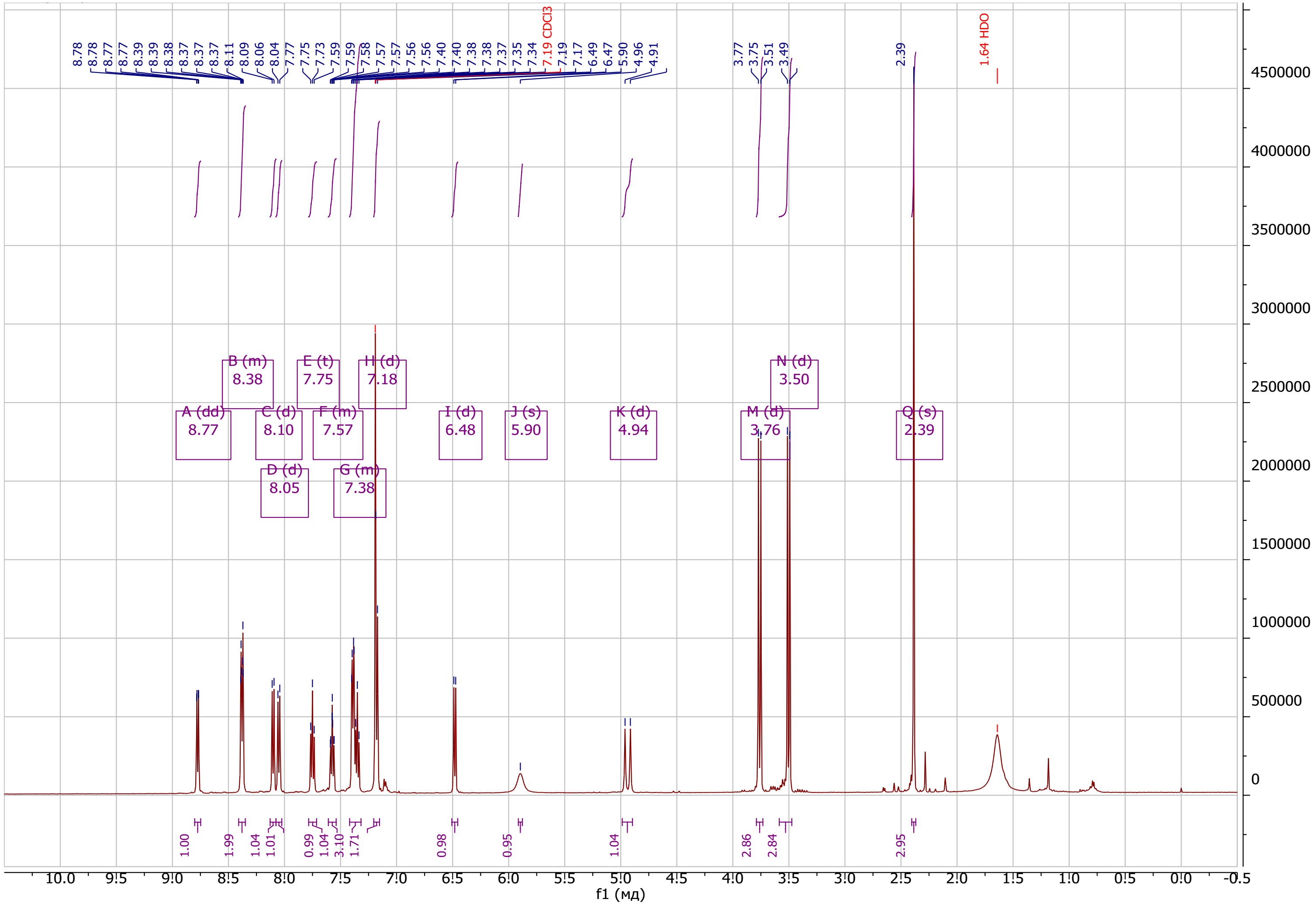

Figure S21. **4b** ^{1}H NMR (500 MHz, Chloroform-d) spectrum.

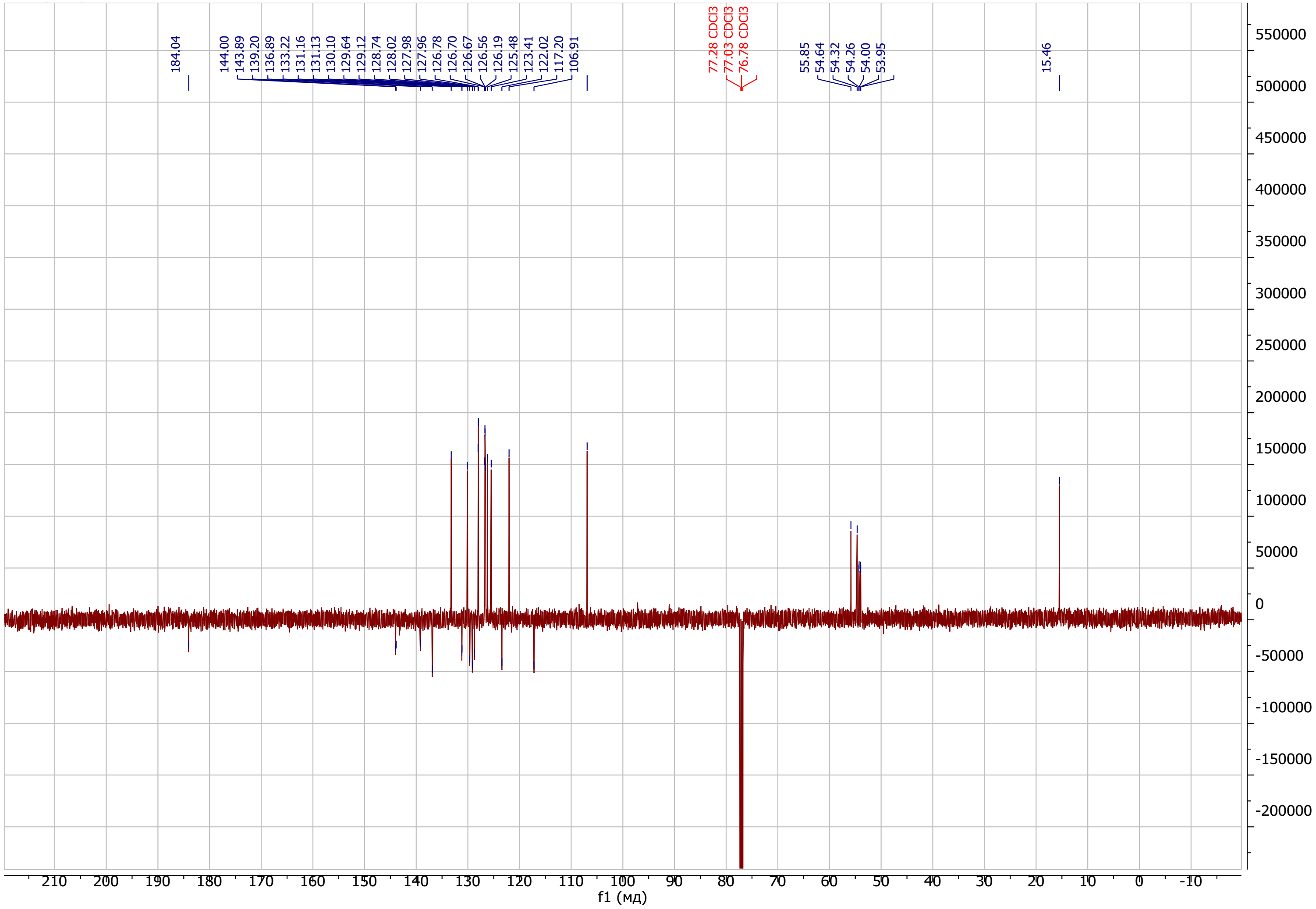

Figure S22. **4b** ^{13}C NMR (126 MHz, Chloroform-d) spectrum.

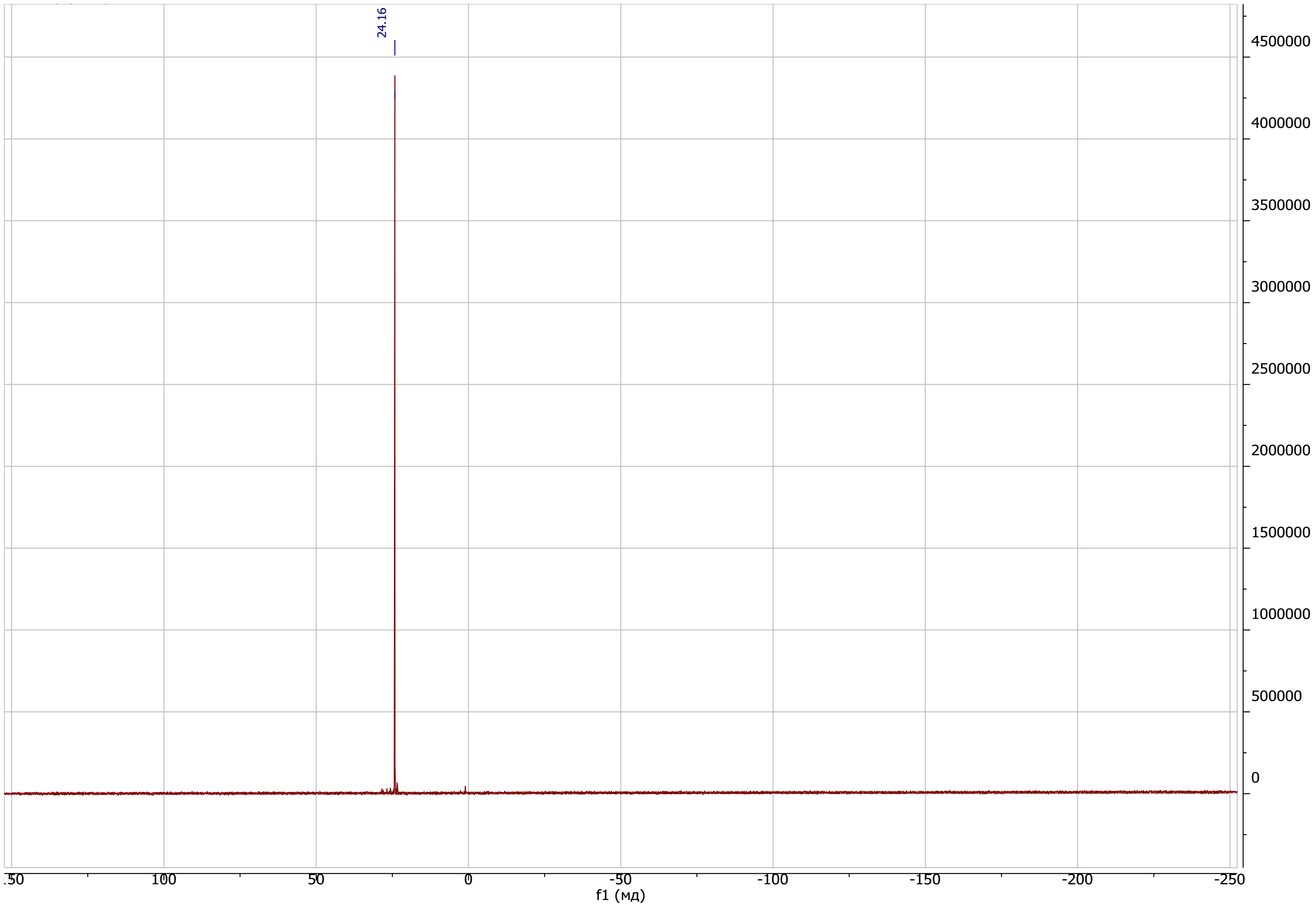

Figure S23. **4b** ^{31}P NMR (202 MHz, Chloroform-d) spectrum.

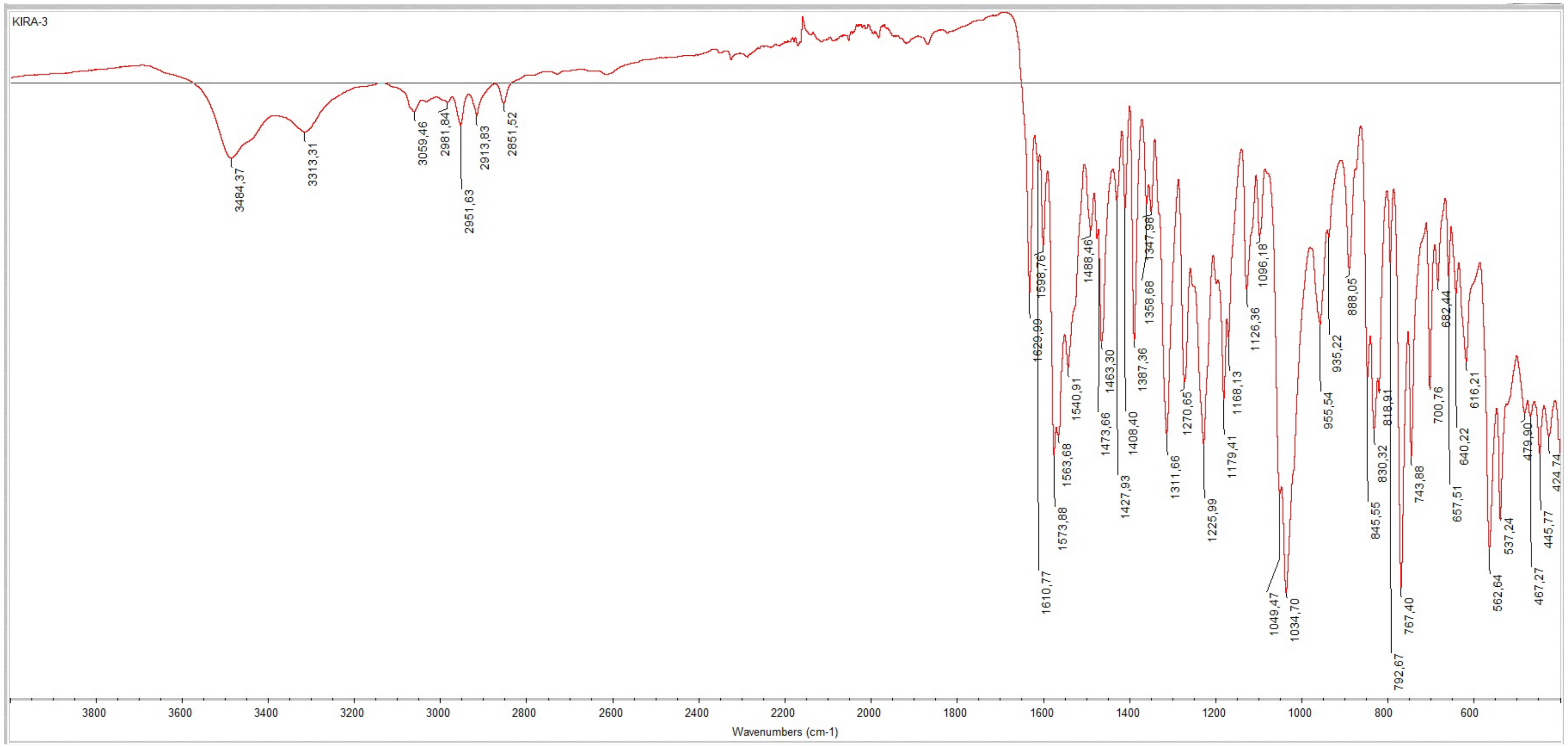

Figure S24. FTIR spectrum of **4b**.

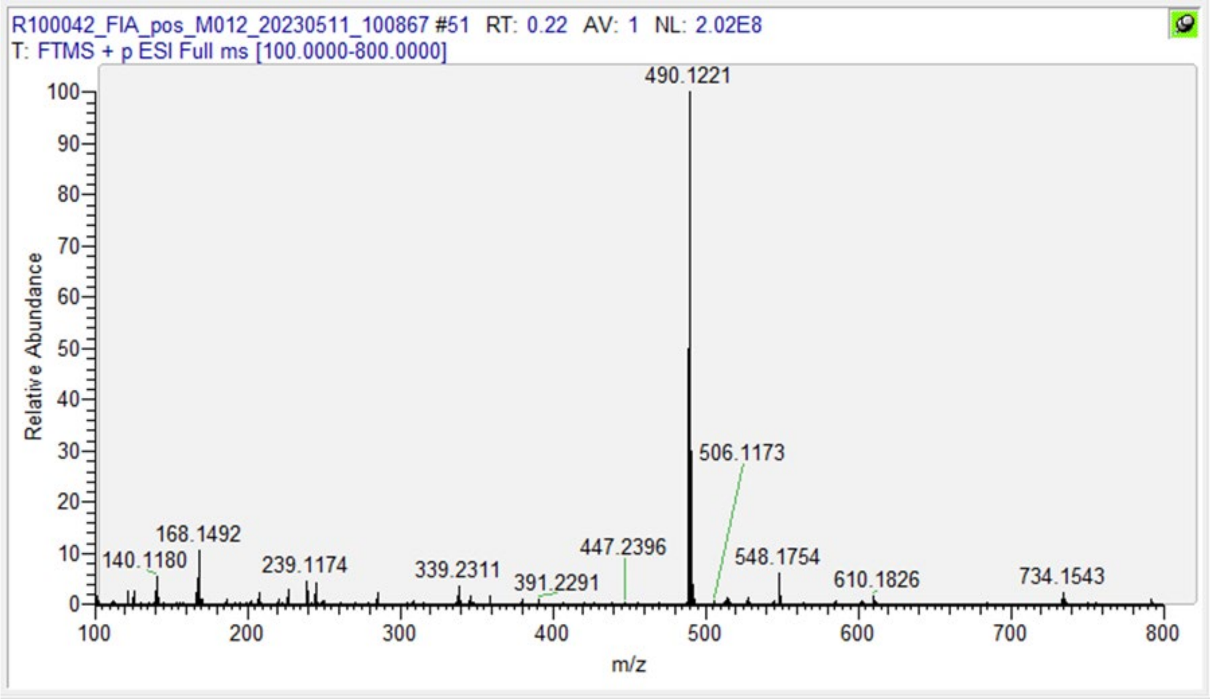

Figure S25. Mass spectrum of **4b** (ESI-FTMS).

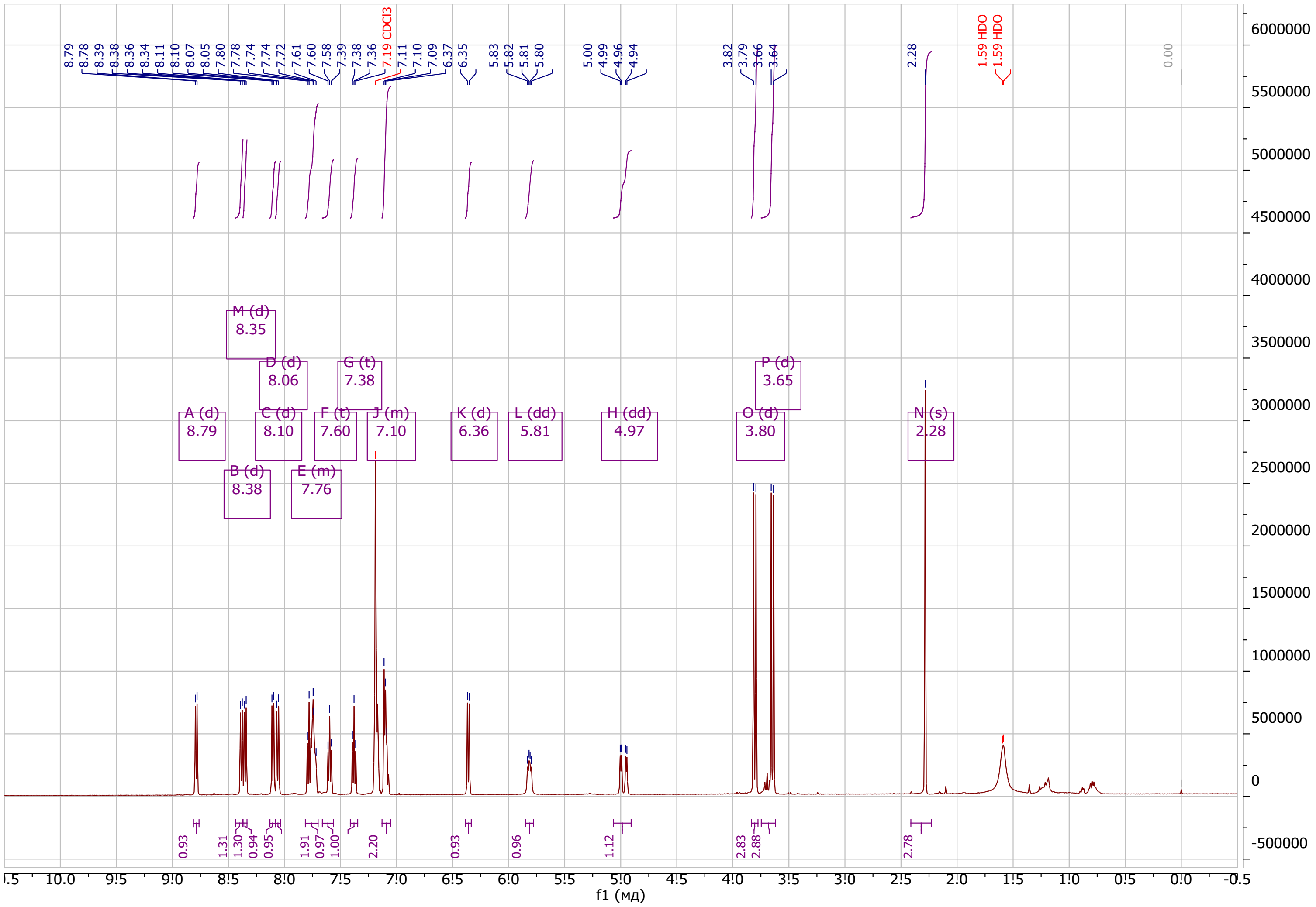

Figure S26. **4c** ^{1}H NMR (500 MHz, Chloroform-d) spectrum.

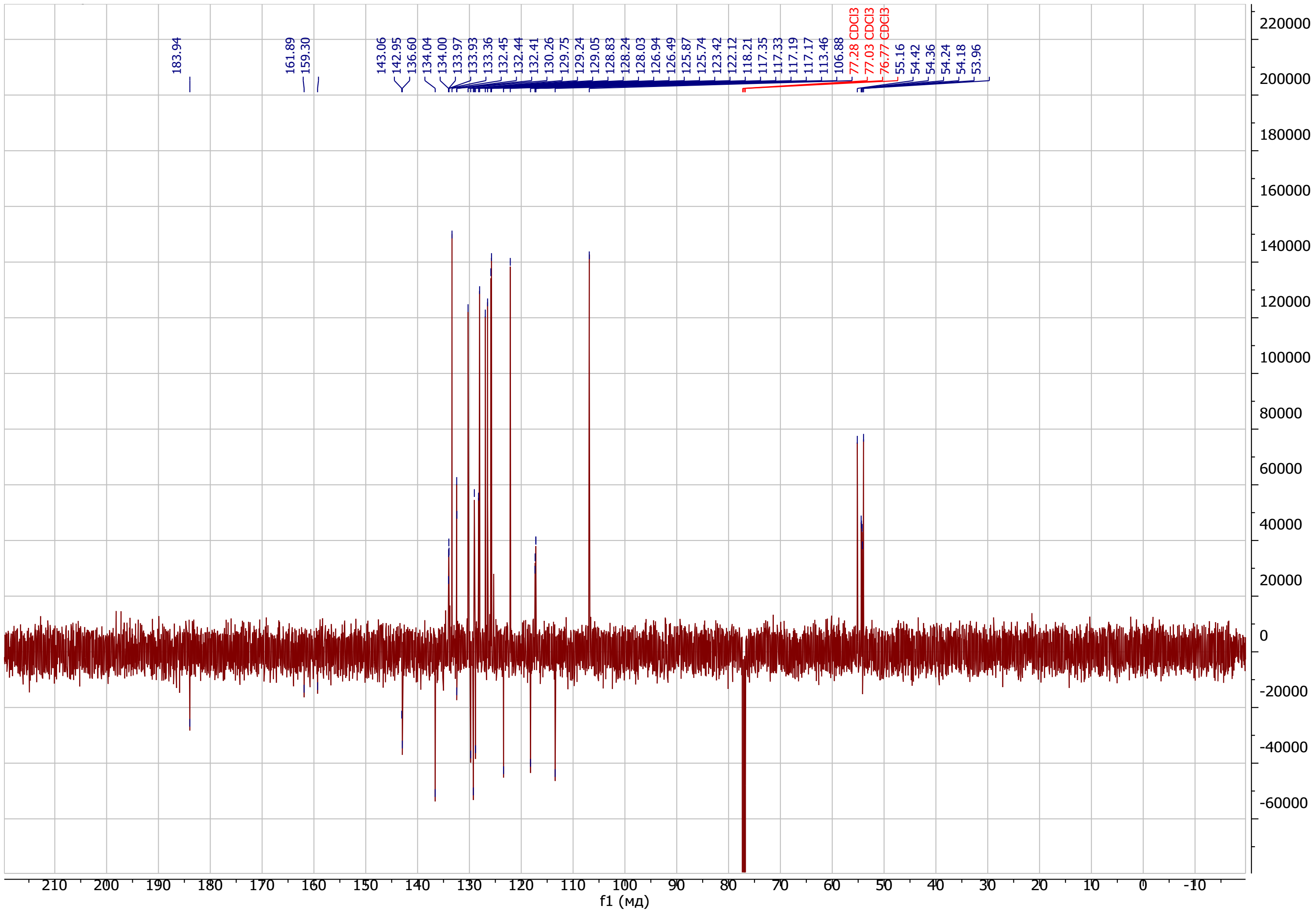

Figure S27. **4c** ^{13}C NMR (126 MHz, Chloroform-d) spectrum.

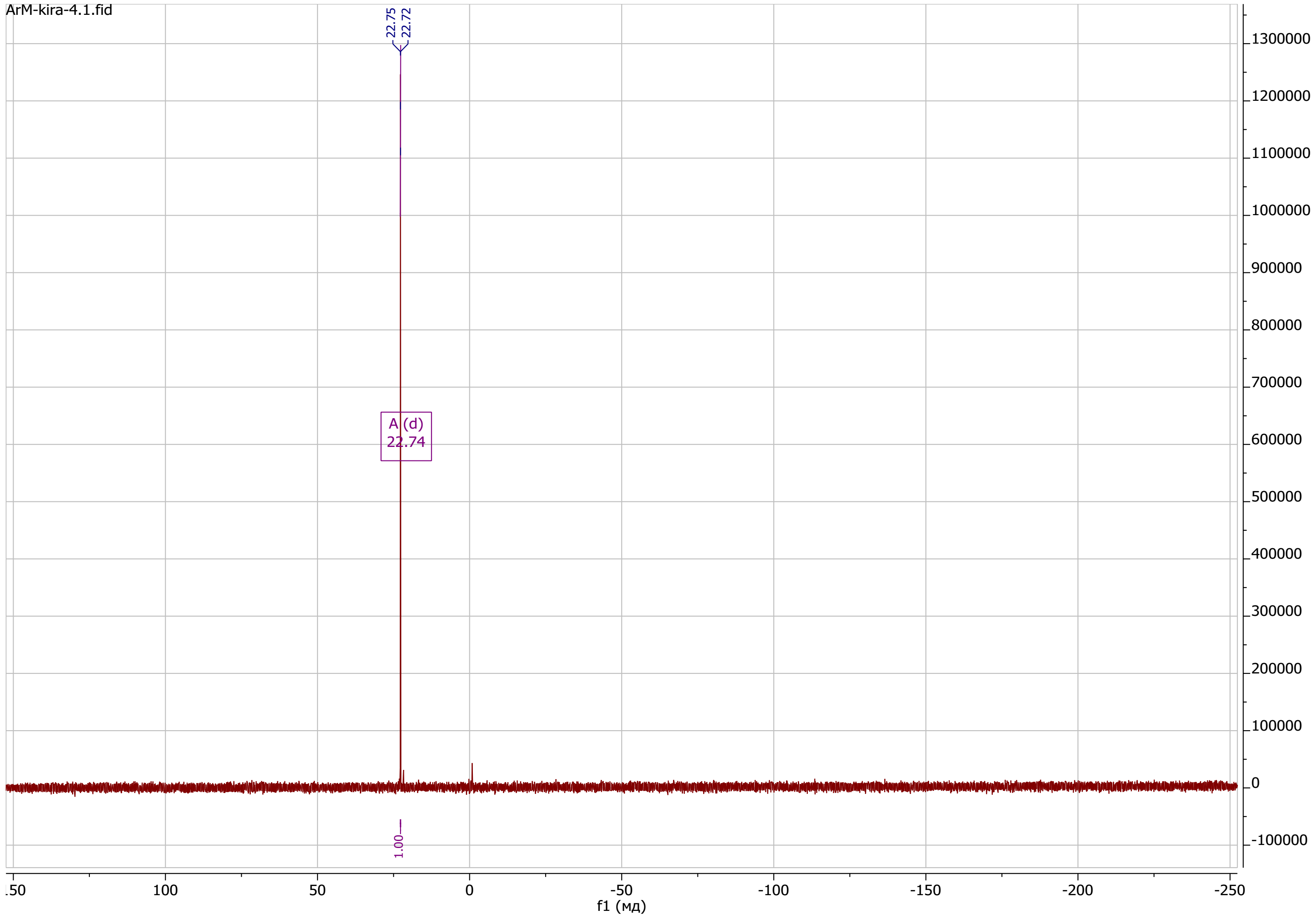

Figure S28. **4c** ^{31}P NMR (202 MHz, Chloroform-d) spectrum.

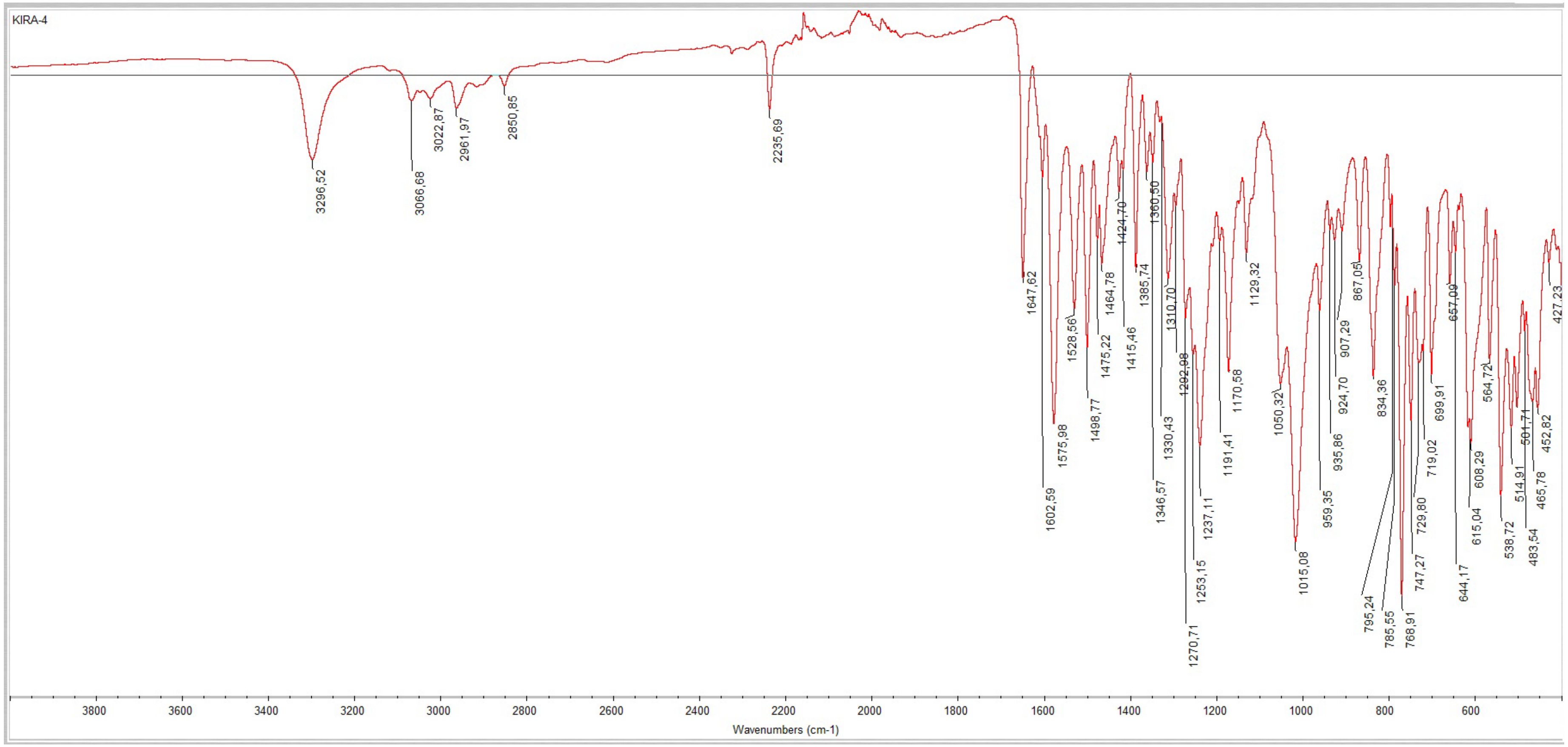

Figure S29. FTIR spectrum of **4c**.

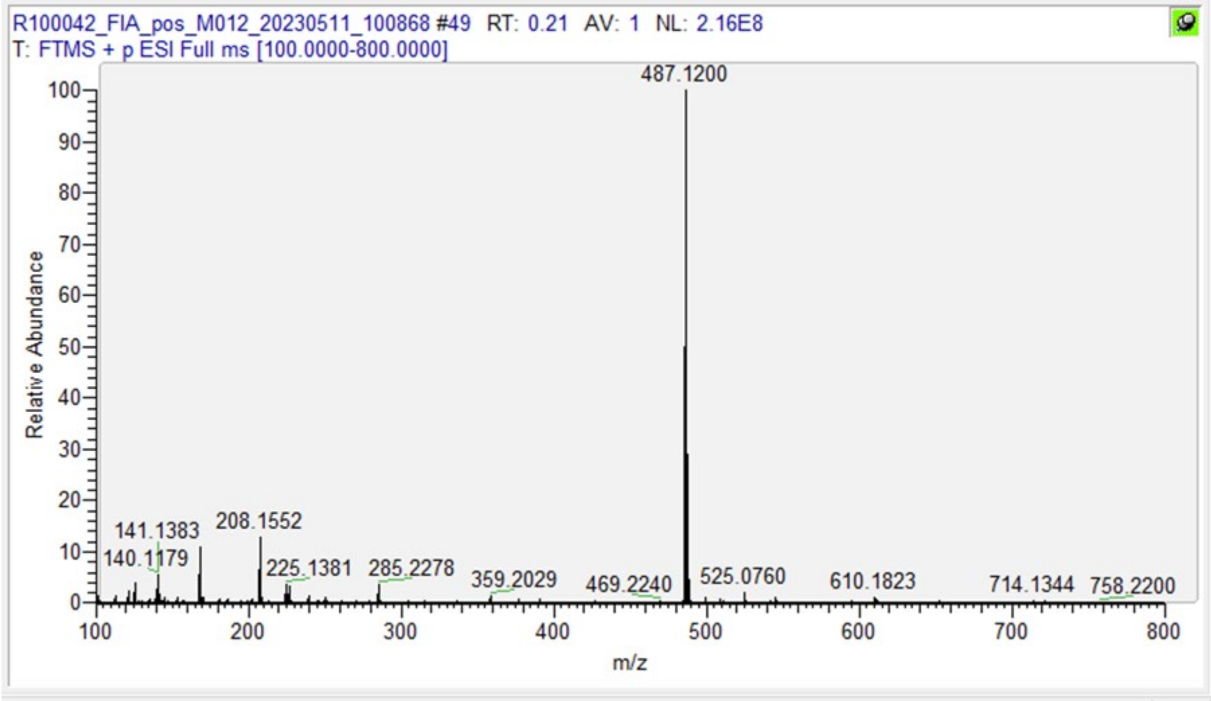

Figure S30. Mass spectrum of **4c** (ESI-FTMS).
